# Self-Ordered Titanium Dioxide Nanotube Arrays: Anodic Synthesis and Their Photo/Electro-Catalytic Applications

**DOI:** 10.3390/ma6072892

**Published:** 2013-07-16

**Authors:** York R. Smith, Rupashree S. Ray, Krista Carlson, Biplab Sarma, Mano Misra

**Affiliations:** 1 Metallurgical Engineering Department, University of Utah, Salt Lake City, UT 84112, USA; E-Mails: york.smith@utah.edu (Y.R.S.); shyama.tum@gmail.com (R.S.R); krista.carlson@utah.edu (K.C.); biplabsarma@gmail.com (B.S.); 2 Chemical Engineering Department, University of Utah, Salt Lake City, UT 84112, USA

**Keywords:** anodization, metal oxide nanotube arrays, photoelectrochemistry, photocatalysis, density functional theory

## Abstract

Metal oxide nanotubes have become a widely investigated material, more specifically, self-organized titania nanotube arrays synthesized by electrochemical anodization. As a highly investigated material with a wide gamut of applications, the majority of published literature focuses on the solar-based applications of this material. The scope of this review summarizes some of the recent advances made using metal oxide nanotube arrays formed via anodization in solar-based applications. A general methodology for theoretical modeling of titania surfaces in solar applications is also presented.

## 1. Introduction

Research focused on the synthesis, characterization, and applications of nanomaterials has recently become a common ground between many scientist and engineers from a variety of fields. Due to the small size of these materials (×10^−9^), they often exhibit exciting electronic, optical, and mechanical properties due to their small geometry. The late Nobel laureate physicists, Richard P. Feynman [1], first envisioned this area of research in his talk “There’s Plenty of Room at the Bottom” at the American Physical Society meeting at Caltech in 1959; however it was not until 1974 that this field was coined the name *nano-technology* by Norio Taniguchi [2].

One class of materials that has recently received considerable interest for a wide variety of catalytic applications is nanostructured transitional metal oxides: in particular, one-dimensional (1D) nanostructured metal oxides such as nanowires, nanorodes, and nanotubes. This class of materials has been widely researched due to their many applications [3]. These materials are generally synthesized using either physical (top down) or chemical (bottom up) techniques. Of the variety of synthesis methods, electrochemical synthesis methods have shown promise to synthesize a number of metal oxide nanostructures [4,5], in particular electrochemical anodization of so-called valve metals and their alloys [6,7]. Through the use of various processing techniques, a wide variety of nanostrucutres can be synthesized with high control.

The synthesis of vertically orientated self-ordered metal oxide nanotube arrays on metal substrates using electrochemical anodization technique have been reviewed [6,7,8], as well as their application in solar energy conversion systems [9,10,11,12,13]. Although several highly ordered nanotubular-type oxide structures of various valve metals and their alloys have been achieved through electrochemical anodization, this review will focus on some of the more recent metal oxide nanotube architectures that have demonstrated solar-based applications, more specifically titania nanotube arrays. A basic methodology for theoretical modeling of titania surfaces for some solar-based applications is also presented.

### 1.1. Electrochemical Anodization

Keller *et al.* [14] first reported the formation of porous anodic alumnia using an electrochemical anodization technique. Subsequently this synthesis method has been extended to several other valve metals and their alloys to form highly self-ordered nanostructures. Despite previous efforts, it was not until the recent works by Hebert *et al.* [15,16] that a quantitative relationship of oxide dissolution and nanoporous film formation on Al and Ti had been established. These works focused on aluminum (Al) and titanium (Ti) but can potentially be applied to other metals. The basis of nanoporous film formation via anodization involves a combination of ionic migration in the formed oxide and stress-driven interface diffusion of metal atoms. A differentiating feature between Al and Ti is that when Al is anodized it generally forms a porous oxide layer, whereas anodization of Ti generally forms a nanotubular oxide layer [17] where separate, individual tubes are formed.

Raja *et al.* [18] suggested that faster generation of cation vacancies by accelerated dissolution and radial transport of vacancies across the pore walls could be the cause of tubular formation when anodizing Ti in fluoride containing electrolytes. Valota *et al.* [19] attributes the separation of nanotubes formed on titania as a result of dissolution of a fluoride rich layer formed at the cell boundaries of the nanotubes; however, this does not explain the separation of nanotubes in fluoride free electrolytes [20,21]. More recently, Su *et al.* [17] proposed a new model based on localized dielectric breakdown during the anodization process. This said mechanism suggests that oxygen filled voids can be generated at the barrier oxide layer of anodic oxides due to localized dielectric breakdown of the oxide. The number and size of such voids increase with the degree of localized dielectric breakdown and the accumulation of these voids at the cell boundary areas causes the separation of neighboring pores [17]. Nevertheless, there is still no generally accepted explanation for metal oxide nanotubular formation via electrochemical anodization process.

**Figure 1:**
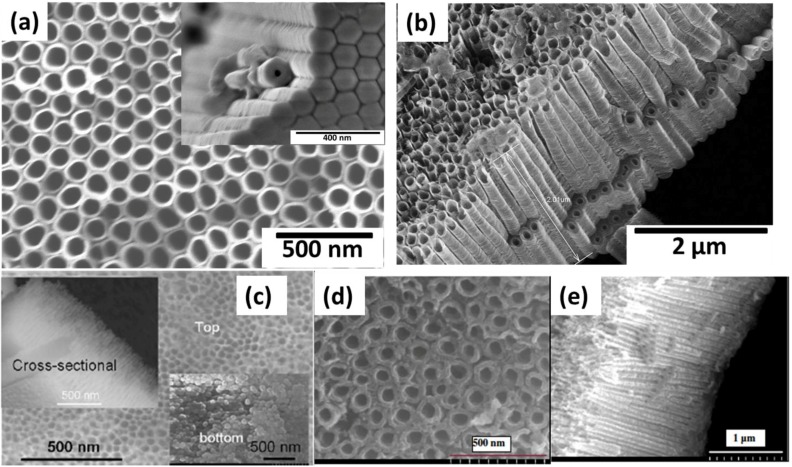
(**a**) Top view SEM micrograph of titania nanotube arrays fromed by electrochemical anodization, with the inset showing the base of the nanotube arrays; (**b**) side vide of the titania nanotube arrays; (**c**) TaON nanotubes formed by nitridization of Ta_2_O_5_ nanotubes (Reprinted with permission from [42], Copyright by The Royal Society of Chemistry); (**d**–**e**) Fe_2_O_3_ nanotubes formed by (Reprinted with permission from [40], Copyright by the IOP Publishing).

Zwilling and co-workers reported the anodization of titanium using fluoride-containing electrolytes in 1999 [22,23]. Nanoporous oxide layers were observed in their studies. Gong *et al.* [24] demonstrated experimental conditions to obtain high-quality and well-ordered T-NTA in acidic fluoride electrolytes. Subsequently, the work of Macak *et al.* [25,26] developed critical parameters required to synthesize well-ordered titania nanotubular arrays (T-NTA) as well as the work of Raja *et al.* [18,27] for various electrolytes. In these methods, a Ti substrate typically in the form of a metal foil (~0.2 mm) is used as an anode during the anodization process. Thin films of Ti (350–1000 nm) have also been successfully used to grow T-NTA. Deposition of Ti on conducting substrates, such as conductive glass (indium tin oxide, ITO) has been carried out using radio frequency sputtering [28]. Anodization of Ti films deposited on Si substrates via DC sputtering [29,30,31], as well as e-beam deposited films [32] have also been reported. T-NTA have also been synthesized using other Ti substrate geometries such as thin wires [33,34,35] and meshes [36,37,38] and curved surfaces [39]. These studies demonstrate the versatility of electrochemical synthesis methods on any substrate geometry. Formation of other metal oxide nanotube arrays that have demonstrated photoelectrochemical applications include hematite nanotubes using iron foil [40,41] and low carbon steel [42], TaON nanotubes from Ta_2_O_5_ nanotubes [43], as well as other Ti-based alloys such as TiN [44], TiPd [45], TiW [46], TiRu [47], TiNbZr [48], among others. Images of various nanotubes are shown in Figure 1.

The electrochemical baths typically used in the anodic formation of metal oxide nanotubes consist of a fluorinated inorganic (e.g., 0.5 M H_3_PO_4_ + 0.14 M NaF) or organic (e.g., 0.2–0.5 wt % NH_4_F + 0.2–10 wt % H_2_O in ethylene glycol/glycerol) based electrolyte. Important parameters in determining the dimensions of the T-NTA include anodization potential (1–150 V, D.C.), anodization time (15 min. to several hours), pH, temperature, and fluoride content. The diameter of the nanotubes is essentially determined by the anodization potential and is linear relationship where an increase in potential results in an increase in diameter. Fluoride content and bath temperature are controlling variables in the wall thickness of the nanotubes. Lower temperatures typically yield thicker nanotubes while higher fluoride content gives thinner nanotube walls. The length of the nanotubes is a strong function of electrolyte pH. Low pH electrolytes results in shorter nanotube lengths regardless of anodization time as a result of self-etching (*vide infra*). Electrolytes with pH~6, such as organic-based electrolytes, can be anodized for longer times and yield much longer nanotubes (up to several microns).

**Figure 2:**
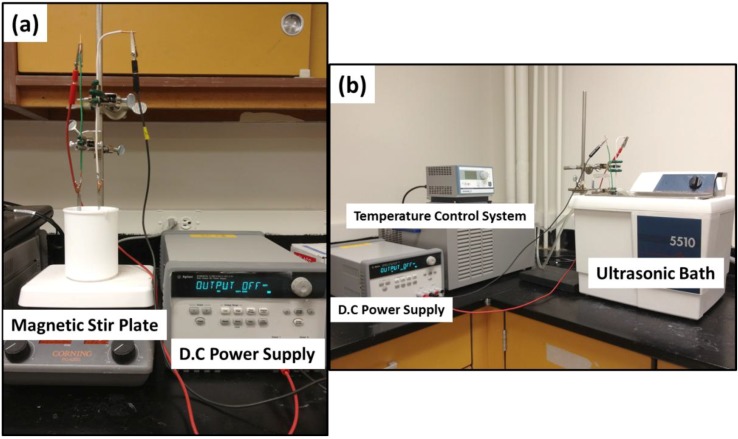
Typical electrochemical anodization setup for the synthesis of metal oxide nanotube arrays utilizing (**a**) magnetic stirring and (**b**) ultrasonication agitation methods. During ultrasonication, to prevent electrolyte heating for prolong synthesis time, the sonication bath temperature is controlled.

A schematic of a typical anodization setup is shown in Figure 2. The experimental setup consists of a two-electrode configuration. The metal to be anodized serves as the anode while a Pt flag of larger area than the anode material serves as the cathode. Potentiostatic anodization is commonly used where the potential is ramped from free corrosion potential to the pre-determined anodization potential. One study [49] examined the galvanostatic anodization on the formation of T-NTA. In this study the authors observed oscillation in the voltage with time, which eventually resulted in unstable oxide films. Other techniques such as pulse voltage anodization [50] have also been applied as well, although similar nanotube morphology is obtained compared to potentiostatic anodization methods.

During the anodization process, the electrolyte bath is commonly mechanically stirred. It was not until the recent works by Sánchez-Tovar *et al.* [51,52] that a detailed study on the effects of hydrodynamic conditions on T-NTA formation was presented. In these studies a rotating disc electrode was used and the effects of flouride concentration (diffusion limited conditions) and Reynolds number on T-NTA were examined. These studies concluded that highly defined nanotubes can be synthesized using defined flow conditions, in particular the nanotube top morphology, which plays a cruial role in photoelectrochemical applications [52]. Other methods of bath agitation such as ultrasonication have also been studied [53]. The use of ultrasonication during anodization, or sonoelectrochemical anodization, results in a more ordered T-NTA morphology. The kinetics of nanotube formation was increased as evident by monitoring the anodic current during anodization. Sonoelectrochemical anodization has also demonstrated enhanced photoelectrochemical performance over photoanodes prepared using magnetically stirred anodization baths [53,54,55]. Recently, we examined the effect of irradiation during anodization of Ti [56]. In this investigation, we found that the nanotube morphology, *i.e*., wall thickness, tube diameter, and sidewall homogeneity, is greatly influenced by irradiation during anodization as well as the electronic properties. It is believed that the photoinduced holes increase the Ti^4+^ cation migration to the surface via Coulombic repulsive forces, which results in greater wall thickness and pore diameter.

#### 1.1.1. Nanotube Formation Stages during Anodization

**Figure 3:**
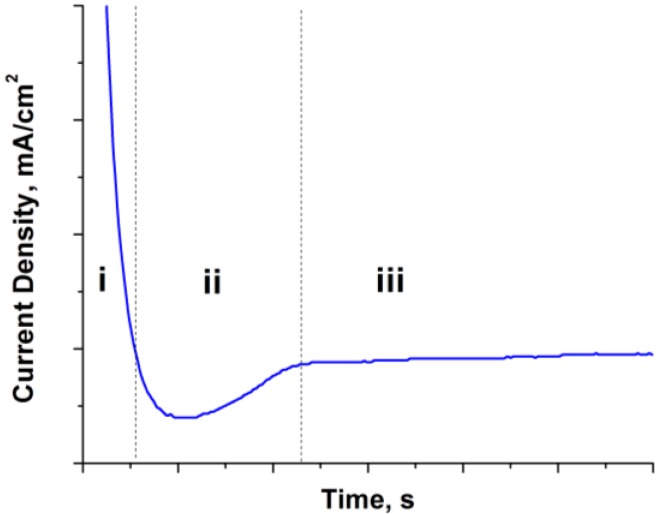
A typical current density *vs*. time plot produced during the anodization process.

Metal oxide nanotube formation in the presence of fluoride ions typically occurs in three stages as outlined by the shape of the anodization current density-time plot as shown in Figure 3: (i) initial oxide barrier layer formation; (ii) pitting/nanopore formation; and (iii) steady-state nanotube growth stage.

In stage (i) a large current density is initially measured at the instant of an applied anodic potential indicating the oxidation of Ti to Ti^4+^. A rapid decrease in current density is observed which is attributed to the formation of oxide layer via hydrolysis reaction


Ti^4+^ + 2H_2_O → TiO_2_ + 4H^+^ (1)

During the hydrolysis reaction, H^+^ ions accumulate and to maintain electroneutrality, F^−^ ions migrate to the H^+^ sites. When a critical concentration is reached at local regions, dissolution of TiO_2_ occurs by the formation of aqueous hexafluorotitanate


Ti^4+^ + 2H^+^ + 6F^−^ → H_2_TiF_6_ (2)

The dissolution reaction of Ti cations creates negatively charged cation vacancies in the oxide which migrate to the metal/oxide interface as a result of the potential gradient across the oxide [57]. The presence of metal-cation vacancies near the metal/oxide interface facilitate the Ti → Ti^4+^ + 4e^−^ reaction as the cations can easily hop to the available vacancy. This event is marked by an increase in current density (stage ii). During this stage, nucleation of nanopores occurs at the oxide surface. Subsequently, steady-state growth of the nanotubular oxide layer is observed when the current density achieves a constant value over time (region iii). It should be noted that the anodic current density during anodization is comprised of two components: the first is current due to the dissolution process at the oxide/electrolyte interface, and the second is current due to the oxidation of titanium at the metal/oxide interface [9]. Moreover, pH of the electrolyte plays a large role in the pore nucleation and growth of the nanotubes as the dissolution rate of the oxide increases with a decrease in pH [58]. Anodization of other metals in a fluorinated electrolyte follows a similar mechanism, for example anodization of Fe to form Fe_2_O_3_ nanotube arrays [40,41,42].

#### 1.1.2. Formation of Complex Nanotube Geometries

Electrochemical anodization allows for growing a variety of tubular- and hierarchical-type nanostructures by changing a few synthesis variables. For example, formation of branching T-NTA is a result from step changes in the voltage during anodization [59,60,61]. However, to form branching tubes, the voltage is not alternated but maintained at the new voltage until chemical and field-assisted reactions (*i.e*., oxidation, ion migration and dissolution) have equilibrated and the branched tube has reached the desired length. Chen *et al.* reported that the number of tube branches could be controlled by reducing the applied voltage by a factor of $1/\sqrt{n}$, where *n* determined the number of branches [60]. Complex multilayer structures were fabricated as adjustments in voltage caused either an increase or decrease in branching. Mohammadpour *et al.* observed the formation of large diameter tubes from the combination of smaller diameter tubes during anodization in low fluorine, viscous electrolytes at high potentials [59]. It was suggested that this structure was the result of the combination of strong capillary forces banding the tubes together and the inhibition of electrolyte between the spaces of adjacent nanotubes. Proposed applications for this hierarchical structure included molecular separation in microfludic techniques and photovoltaics owing to the higher surface area and better charge separation at the “core-leg” interfaces [59].

Bamboo T-NTA structures can be formed through electrolyte chemistry modifications as well as alternating applied bias. The banded, bamboo-like structure can occur in aqueous or high water content glycerol electrolyte solutions [62,63]. However, glycerol solutions containing glycerol/H_2_O ratios higher than 9:1 do not exhibit nodes due to the lack of periodical current fluctuations at the T-NTA surface. No nodes are observed when ethylene glycol replaces glycerol at similar ratios. This observation was attributed to the higher diffusion constant of the EG, which reduced the fluctuations in current density during tube formation. More control over the node thickness and frequency is provided by the application of an alternating bias in an ethylene glycol based solution [64]. Bamboo structures are often used in applications such as DSSC where maximized dye loading is necessary. Although the stratification between the nanotubes causes a longer random path for photogenerated charges, improvements in incident photon to current efficiency (IPCE) are still observed due to the additional surface area provided by the nodes. The bamboo rings allow for a higher rate of dye loading due to extra surface area. The extra space introduced between the tubes due to the rings also allow dye molecules to cover the exterior of the NT walls [64]. Kim *et al.* reported that bamboo structures with 70 nm spacing between the compact nodes provided the largest increase in photoactivity [64].

Hierarchical structures of T-NTA such as nanolace-type structures atop the nanotube arrays can be achieved by alternating voltage technique [65] or through surface treatments [66,67,68]. Double walled T-NT have also been synthesized using an ionic liquid fluoride solution in ethylene glycol [69]. The common synthesis technique adopted for growing hierarchical-type T-NTA involves a two-step anodization process. First, T-NTA are grown on a Ti substrate and subsequently removed via ultrasonication for a long duration, leaving a patterned Ti substrate. The as-formed substrate is then subject to a second anodization, resulting in smooth nanotubes with a nanoporous/nanolace top layer. Recently we have demonstrated a simple surface etching treatment such that T-NTA can be synthesized by a single anodization process at constant voltage [68].

#### 1.1.3. Crystallization of Nanotubes

The as-anodized oxide layer formed is amorphous in nature. For many applications, the amorphous crystal structure is too disordered and does not have desirable electronic properties. Generally, crystallization is achieved through thermal treatment at 350–500 °C in a variety of atmospheres. In the case of T-NTA, of the three crystal structures of titania (rutile, anatase, and brookite) a mixture of anatase and rutile are the primary crystal structures obtained after thermal annealing in either air or oxygen whereas annealing in inert (N_2_) or a reducing atmosphere (H_2_/N_2_) result in primarily anatase phase titania [70]. For solar-based applications, predominantly anatase phase titania is most desirable [71]. Although anatase and rutile are the primary crystal structures obtained through thermal treatments, a recent study has demonstrated the formation of brookite T-NTA between 470 and 500 °C in air [72]. Other methods examined to form crystalline T-NTA via non-thermal routes are by hydrothermally treatments [66] or by soaking them water at room temperature for an extended period of time (days) [73]. Despite the perceived benefits of such methods to obtain crystalline T-NTA, for solar-based applications, thermal treatment results in the most photoactive oxide layer for T-NTA [74].

### 1.2. Surface & Bulk Properties of TiO_2_

Although TiO_2_ naturally occurs in several different polymorphic forms, anatase and rutile are the most commonly studied phases for nanomaterials used in photocatalyic applications [75]. The basic structure of both anatase and rutile contains titanium in six-fold coordination with oxygen [75,76]. Differences between the two phases arise from the connection of the octahedrally coordinated Ti cations; anatase octahedra share four edges and are connected in staggered chains parallel to the [221] direction, while the octahedra in rutile form chains along [001] direction with only two shared edges [76]. Polymorph stability is dependent upon synthesis method and crystallite size [75,76,77,78]. At macroscope levels, rutile is the most thermodynamically stable phase under ambient conditions. However, as crystallite size drops below 13 nm, anatase becomes the stable phase [76,79]. This phase change suggests that many of the ordinary physics and chemistry rules of bulk materials no longer apply at the nanolevel, allowing their properties to differ substantially [78].

The relationship between the bulk and surface chemistry of a material becomes apparent when examining the surface charge that develops when it is placed in an aqueous solution. When immersed in an aqueous solution, the charge that develops on an oxide surface is mainly dependent upon the electronegativity of the cations in the material and the solution pH [80]. However, these values have been found to vary depending on polymorph, different crystallographic planes, crystallite size, as well as synthesis and measurement methods. For example, Mandzy *et al.* [81] reported the isoelectric point (IEP) of different sized nanoparticles of antase and rutile: 5 nm anatase–pH 4, 25 nm anatase–pH 5.5 and 10 nm rutile–pH 3.2. As commercially available materials, the synthesis method was not listed; however, the 5 nm anatase value differed from the pH~5.5 reported by Penn *et al.* [76] where 5 nm particles were created via a sol gel method. This discrepancy is most likely the result of the technique used to obtain the IEP value. In Mandzy’s study, the IEP was measured using a zetameter, while Penn relied on the gelling of suspensions. Difference in crystallite size could account for the discrepancy between Mandzy’s 10 nm rutile particles exhibiting an IEP of pH 3.2 and 2 mm diameter particles formed by Bullard *et al.* [82] which had an IEP of pH 5.2. Bullard also demonstrated the variation between crystallographic planes with IEP ranges for the (100), (110) and (001) surfaces of rutile as 3.2–3.7, 4.8–5.5, and 5.5–5.8, respectively.

Differences between polymorphs and crystal surfaces can be explained through surface chemistry. Bullard theorized that differences between Ti and O coordination caused the variation in IEP. It was suggested that higher IEP values for the (001) surface were due to stronger and more numerous Lewis base sites. The increased quantity and strength of these electron-donating oxygen positions facilitated more adsorption of solvated hydrogen ions. The (001) surface is also more open than the (100) and (110) plane, promoting high charge mobility, and hence would be the preferred orientation in electrochemical experimentation.

The relationship between surface acidity and nanotube structure was examined extensively by Kitano *et al.* [83]. In this study, Fourier transform infrared (FT-IR) spectroscopy was used to examine TiO_2_ nanosheets and nanotubes with similar crystal structures. Nanotubes and nanosheets were found to possess both Bronsted and Lewis acid sites; however, nanotubes were found to exhibit higher Bronsted acidity than the nanosheets. It was suggested that the higher concentration of bridging OH *vs*. terminal OH groups found on nanosheets, and the distortion of the Ti octahedra, allowed for nanotubes to have higher catalytic activity than the nanotsheets. Still, few surface aciditiy studies have been performed on titania nanotubes formed via anodization, leaving this area in need of further investigation. However, many common surface modifications, along with their effect on photocatalytic reactions, are discussed in the following sections.

### 1.3. Photocatalysis and Photoelectrochemistry

The photovoltaic effect is a phenomena in which a voltage or electrical current is created in a material by being exposed to electromagnetic radiation, light energy more specifically, and is the basis of semiconductor photocatalysis and photoelectrochemistry. The French scientist Alexandre-Edmond Becquerel first reported witnessing this effect in 1839 [84]. Several advances between the 1950’s and 1970’s improved the understanding of electrochemical interactions between semiconductor-liquid interfaces, namely by Gerischer, Memming, and Williams, among others [85,86,87]. These studies resulted in establishing a fundamental understanding of the semiconductor-electrolyte interface including kinetics and energetics of charge transfer across the semiconductor-electrolyte junction. Despite these early fundamental studies, it wasn’t until the 1970’s works’ by Fujishima and Honda that first demonstrated the potential application of photoelectrochemical systems for energy conversion and storage [88,89]. Their initial studies demonstrated that the oxidation of water could be carried out at a less negative potential compared to the standard redox potential when a titania surface is irradiated with light energy greater than the material’s band gap. These results initiated the realization that sunlight could be used to split water into hydrogen and oxygen, a process commonly referred to as photoelectrolysis [90].

Solar energy is an attractive energy source for energy conversion and storage as nearly enough solar energy strikes the earth in one hour to meet nearly all our global energy demands for an entire year [91]. Over the years there have been numerous studies on semiconductor photocatalytic and photoelectrochemical systems for hydrogen generation (water splitting reaction) of which have also been extend to many other areas such as electrochemical photovoltaics [92,93,94], wastewater remediation [95,96], antibacterial/self-cleaning surfaces [97,98], and fuel synthesis [99], among others.

**Figure 4:**
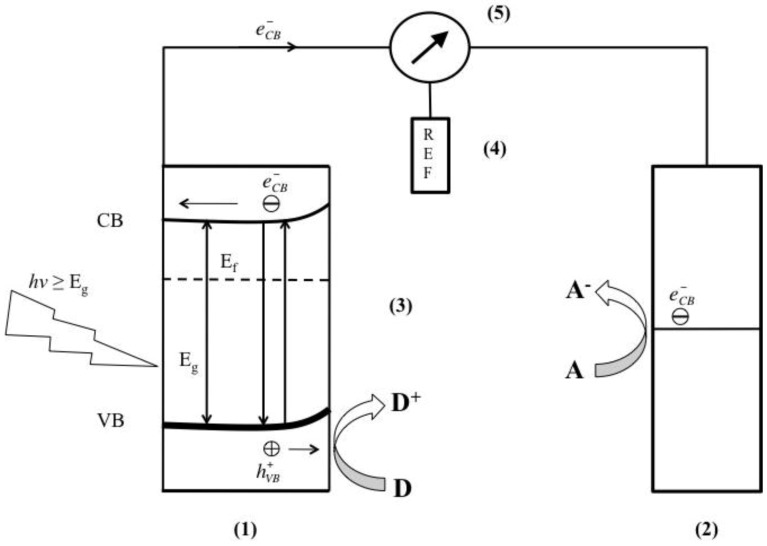
Schematic of a liquid junction photo-electrochemical cell for a p-type semiconductor for the reduction of carbon dioxide to various products. A similar setup is used for n-type cells as well. The anode (1) is the photoactive material; (2) is the cathode; (3) a conducting electrolyte; (4) reference electrode; (5) potentiostat. The Fermi level of the semiconductor is denoted by E_f_. Figure adapted from Reference [100].

Figure 4 shows a schematic of a liquid-junction photoelectrochemical cell. It consists of a photoanode, a counter electrode cathode, and a reference electrode. In a typical PEC setup, semiconductors utilize light irradiation to promote reactions on the surface of the electrodes within the system. This example considers n-type semiconductors, as they have generally shown better stability. The principles and applications of p-type materials are generally similar to n-type materials. Oxidation of the electrolyte (e.g., H_2_O) occurs at the anode and reduction occurs at the cathode.

An innate property that makes semiconductors useful for PEC is their discrete quantum states of electrons. Unlike metals that have a continuum of electron states (bands), semiconductors exhibit an electrical resistivity, or rather an energy band gap (E_g_) that extends from the top of the filled valance energy band (VB) to the bottom of the vacant conduction energy band (CB). In general, when a semiconductor surface is exposed to light radiation (hν ≥ E_g_) electron hole pairs (*e*^˗^_CB_ + *h*^+^_VB_) are generated, represented by Equation 1 with heat generation for the reverse reaction.

*Photocatalyst* → (*e*^˗^_CB_ + *h*^+^_VB_) (3)

*photocatalyst* + (*e*^˗^_CB_ + *h*^+^_VB_) → Δ(heat) (4)

**Figure 5:**
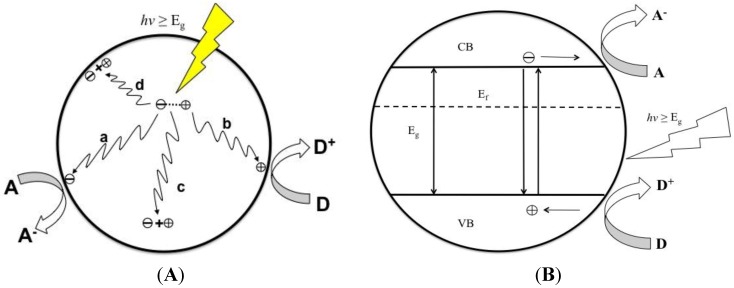
(**A**) Schematic of photoexcitation in a solid volume followed by recombination events. Paths a and b represent surface scavenging events while paths c and d respresent inner volume and surface recombination events. Figure adapted from Reference [100]; (**B**) A photocatalytic system where the energy bands (VB and CB) act as the anode and cathod in a PEC system. Figure adapted from Reference [100].

Upon photo-excitation of an electron from the VB to the CB, the *e*^˗^_CB_ and *h*^+^_VB_ pairs can follow several pathways (Figure 5A). Ideally, in a PEC cell the photo-generated *e*^˗^_CB_’s are driven to the external circuit and subsequently to the cathode with a small bias potential. The *h*^+^_VB_’s can then migrate to the surface of the semiconductor to oxidize any absorbed molecules or solvent on the surface. The ability for a photocatalyst to carry out redox reactions and facilitate effective and efficient charge transfer is predisposed to two criteria are often required: (i) effective charge separation; and (ii) the catalysts’ ability to absorb reacting species on its surface. At the surface of the semiconductor, electrons can also be donated to an acceptor species and likewise the holes can migrate to the surface to combine with electrons from a donor species as well (Figure 5, path a and b). However, the recombination of electrons and holes prevents them from transferring to the surface to react with absorbed species. Recombination can occur within the volume of the semiconductor or on the surface of the semiconductor (Figure 5, path c and d). The rate at which charge transfer occurs depends up the position of the bands and the redox potential of the absorbed species of interest.

For photocatalytic systems, Bard’s concept [101] can be applied. This concept suggests that semiconductor particles can act as a short-circuited PEC cell by providing both oxidizing and reducing sites for the reaction (Figure 5B). When comparing the two systems, the photocatalytic system is simpler and easy to construct. Some important factors that should be met for optimal performance of a photocatalytic include:

- The redox potential of the photogenerated VB hole should be sufficiently positive for the hole to act as an acceptor;
- The redox potential of the photogenerated CB electron should be sufficiently negative for the electron to act as a donor;
- Photocatayst should be economically available and be environmentally inert;
- Photocatalyst should be stable in a wide pH range and in a variety of electrolytes.

## 2. Aqueous Photocatalytic Mineralization

The ability to efficiently and inexpensively degrade organic pollutants and inactivate bacteria is important as industrial, agricultural and residential waste streams continue to increase with the growing world population. Titania substrates have been studied extensively as catalysts in photo-reactive oxidation reactions for the decontamination of groundwater and wastewater streams. Of the metal oxide nanotube arrays formed by electrochemical anodization, T-NTA is the most studied material. In comparison to titania nanoparticle systems, T-TNA provide a number of benefits. For example, post-treatment recovery of titania nanoparticles from slurry reactors is inefficient and expensive [102]. Immobilizing titania nanoparticles has been carried out on conducting substrates to counter this problem [103,104,105,106]. However, when the titania is cast as films a significant reduction in surface area occurs causing degradation times to be prolonged [107,108]. More efficient pollutant degradation using T-NTA over compact titania surfaces can be attributed to fewer interfacial grain boundaries which promotes better charge separation and improved redox activity [109]. The following section will address the photooxidative mineralization of organics, the inactivation of microorganisms, as well as carbon dioxide reduction to fuels.

### 2.1. Organic Pollutants

**Figure 6:**
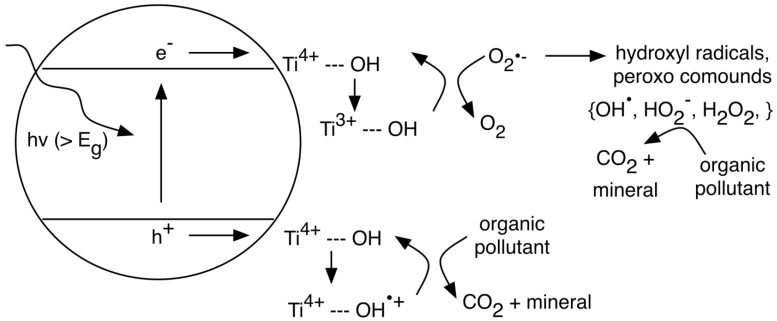
Photooxidative degradation of organic pollutants through interaction with surface bound hydroxyl radicals.

Titania photooxidation of organic pollutants can occur via interactions with oxidizing radicals and/or photogenerated holes [110,111]. The position of the TiO_2_ band edges, as well as the redox potentials of the pollutant are crucial in determining what type of PC reactions will take place. Degradation in aqueous solutions is typically the result of interactions with reactive radical ions. Under irradiation (hν > E*_g_*), photogenerated charges react with H_2_O to form superperoxo (O_2_^•−^) and hydroxyl radicals (OH^•^). Interaction of the organic (e.g., dye molecule) with free or surface bound radicals causes mineralization as illustrated in Figure 6. Complete destruction of the organic results in CO_2_ and mineral acids and can be expressed as follows [111,112,113]:

TiO_2_ + hν → TiO_2_ (e_CB_^−^ + h_VB_^+^) (5)

TiO_2_ (e_CB_^−^ + h_VB_^+^) →TiO_2_ + heat (6)

TiO_2_ (h_VB_^+^) + H_2_O →TiO_2_ + H^+^ + OH^•^ (7)

TiO_2_ (h_VB_^+^) + OH^•^ →TiO_2_ + OH^•^ (8)

TiO_2_ (e_CB_^−^) + O_2_ →TiO_2_ + O_2_^•−^ (9)

O_2_^•−^ + H^+^ → HO_2_^•^ (10)

Dye + OH^•^ → degradation products (11)

Dye + h_VB_^+^ → oxidation products (12)

Dye + e_CB_^−^ → reduction products (13)

**Figure 7:**
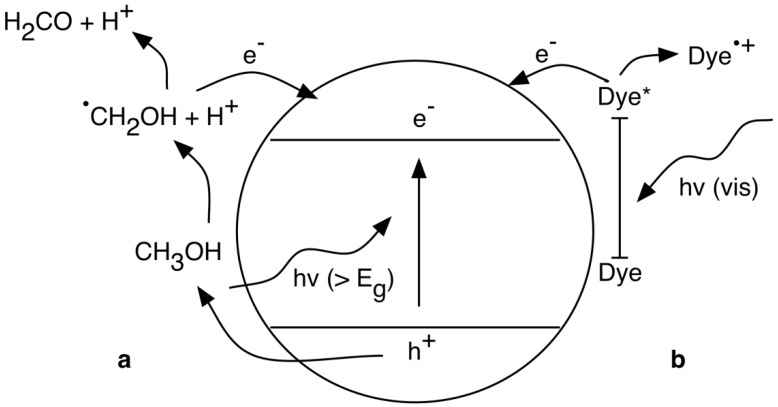
Schematic of the (**a**) *photocurrent-doubling effect* where the absorption of one photon by methanol can lead to the generation of two electrons in the TiO_2_ CB; and (**b**) dye sensitization of TiO_2_ leading to an injection of an electron into the conduction band.

Photooxidation can also occur through direct charge transfer between TiO_2_ and the organic pollutant. The effectiveness of TiO_2_ to act as a photocatalyst in these degradation reactions is due to the position of the energy band on the electrochemical potential scale [110,111,114,115]. The position of the conduction and valence band edges becomes even more important in situations where it is thermodynamically favorable for the photogenerated charges to be directly scavenged by the pollutant [114,115,116,117]. An example of this process is the oxidation of methanol where charges are directly transferred from the TiO_2_ surface to methanol and its degradation products [114]. The primary oxidation product of methanol, the methoxy radical, is a strong reducing agent in this situation in that it is able to transfer another electron to the conduction band (CB) of TiO_2_. This process, illustrated in Figure 7 (reaction a), is an example of the *photocurrent-doubling effect* [114], as two electrons in the CB have been generated upon the absorption of one photon. The band-edge position of TiO_2_ is suitable for many PC activities; however, it is often desirable to reduce the magnitude of the band gap to make visible light-induced reactions thermodynamically favorable.

The degradation reaction pathways described above are different for photosensitized oxidation reactions, where the photo-process is initiated through visible light absorption of a sensitizer [112,114]. This process, shown in Figure 7 (reaction b) with a typical dye sensitizer, can be considered *indirect photocatalysis*, as visible light excitation of the sensitizer leads to electron injection from the excited molecule into the conduction band of the TiO_2_. The cationic dye radicals generated from reaction are as follows:

Dye + hν → Dye*
(14)

Dye* + TiO_2_ → Dye^•+^ + TiO_2_ (e_CB_^−^) (15)

TiO_2_ (e_CB_^−^) + O_2_ → O^•−^ + TiO_2_ (16)

Dye^•+^ → degradation products (17)

Dye radicals can be completely mineralized through reactions with hydroxyl radicals (Equations 14 and 15) or interact with peroxo compounds (O_2_^•−^; HO_2_^•^ or HO^•−^) to form intermediates that eventually decompose to CO_2_ and H_2_O (Equations 20–24):

Dye^•+^ + OH^−^ → Dye + HO^•^ (18)

Dye + 2HO^•^ → H_2_O + oxidation products (19)

O_2_^•−^ + H^+^ → HO_2_^•^ (20)

HO_2_^•^ + H+ + TiO_2_ (e_CB_^−^) → H_2_O_2_ + TiO_2_ (21)

H_2_O_2_ + TiO_2_ (e_CB_^−^) → HO^•^ + HO^−^ + TiO_2_ (22)

Dye^•+^ + O^•−^ → DO_2_ → degradation products (23)

Dye^•+^ + HO_2_^•^ (or HO^•^) → degradation products (24)

### 2.2. Surface Charge

The rate of photomineralization is highly influenced by solution pH, as it alters the surface charge of TiO_2_ and the ability of oppositely charged species to adsorb to its surface [114,116,118,119,120]. The effect of this parameter is dependent upon the ions present in solution. In acid or basic conditions, the surface of titania can become positively or negatively charged, respectively. The zero-point charge pH (pH_ZPC_) of titania depends on the synthesis method, but has been reported between 6 and 7 for titania nanotubes [119,121]. Thus, for pH values less then pH_ZPC_ the surface is positively charged due to H^+^ ions and for pH values greater than pH_ZPC_, the titania surface becomes negatively charged due to OH^−^. In the case of chlorophenols, which have a negative charge at low pH, Ku *et al.* [118] reported that low pH solutions encourage PC degradation because TiO_2_ carries a net positive surface charge. However, reports of higher decomposition rates with high pH values is also reported [119]. Increased photooxidation rates in increasingly more basic medium can be attributed to an increase in hydroxyl radical formation as the hydroxide ions scavenge the holes to form hydroxyl radicals (OH^−^ + h^+^→ OH^•^).

The presence of interfering adsorbing species, such as inorganic anions (*i.e*., chloride, sulfate, nitrate), can diminish photo-induced organic mineralization [116,119]. The inhibitive effect is due to the occurrence of competitive adsorption onto the TiO_2_ surface. Chlorine ions are strong retarders of photooxidation as it not only readily adsorbs to TiO_2_, but can also scavenge holes and hydroxyl radicals. Inorganic anions are charged species, and as such, the extent of interference is dependent on solution pH. Liang *et al.* reported on both the effect of Cl^−^ ions on 2,3-dichlorophenol (2,3, DCP) decomposition rate, as well as the effects of NO_3_^−^, H_2_PO_4_^−^ and SO_4_^2−^ [119], in solutions of pH 5.3. As the Cl^−^ ion concentration increased from 0 to 0.1 M, the decomposition of 2,3-DCP after 300 min decreased from 93% to 64%, respectively. Photo-decomposition rates using 0.05 M anion concentrations of Cl^−^, NO_3_^−^, H_2_PO_4_^−^ and SO_4_^2−^ also exhibited lower photo-decomposition rates. As the surface charge of TiO_2_ is positive at a pH of 5.3, the divalent SO_4_^2−^ ion has the highest inhibitory effect on decomposition due to its stronger competitive adsorption. Similar to Cl^−^, SO_4_^2−^ can also inhibit photodegradation through the entrapment of positive holes and/or hydroxyl radicals. This observation changes, however, as the pH is increased to 6. Brugnera *et al.* reported that as the surface charge of the TiO_2_ became more neutral, SO_4_^2−^ ions had little effect on the degradation of Bisphenol A [116]. However, the presence of NO_3_^−^ cut the decomposition performance nearly in half. It was suggested that this inhibitory effect occurred due to nitrate absorption of UV light, causing the transformation from nitrate to nitrite, and subsequently blocking irradiation from the TiO_2_ surface.

The reduction of molecular oxygen at the surface of TiO_2_ can act as a rate-limiting step in the photocatalytic degradation of organic pollutants [111,119]. When molecular oxygen is present at the surface of TiO_2_, it can act as an electron acceptor and remove photogenerated electrons. As this transfer process is slow, adsorbed oxygen should be excess of the electrons to maximize this transfer rate. While levels below ~8 mg/L of dissolved oxygen show lower degradation rates, concentrations above this level do not increase it significantly [119]. Dissolved oxygen levels are also affected by temperature, tending to decrease with increasing temperature. Although an increase in solution temperature leads to an increase in species oxidation rate, overall decomposition will be counteracted by the lower oxygen levels and adsorption isotherms [111].

### 2.3. Nanotube Geometry

Dimensional factors of the nanotubes, such as pore diameter, tube length and wall thickness, have also been reported to play a role in organic photodegradation [122,123,124], although varying results are found in the literature. Longer nanotubes can absorb more light; however, the depth of incident photon penetration is limited. Therefore, once a certain length range has been exceeded, further improvement in photocatalytic activity will no longer be observed. Another disadvantage is the limitation of organic diffusion inside of the nanotubes, which is not significantly affected by stirring [122]. Larger nanotube diameters were also found to exhibit better photodegradation, as wider pores provided improved accessibility to the organics and a lower probability of electron-hole recombination [124]. There was an observed upper limit on this increased photoactivity, with pores greater than 75 nm showing little improvement in organic decomposition. Wall thickness was reported to be the most significant parameter, as thinner walls are more efficient at channeling photogenerated charges to the T-NTA surface in PC reactions [122]. This finding is supported by the superior photodegradation capabilities of nanowires (or nano-grass) over nanotubes due to an increase in the charge transport along the narrow tube walls [125].

Photocatalytic degradation is also sensitive to titanium substrate geometry. Titania nanotubes formed on wires or mesh provide a 3D structure where improved photodecomposition occurs due to the tubes capacity to not only absorb incident light, but reflected and refracted light as well [33,126]. Nanotube coated wires also showed superior degradation capabilities over Pt loaded T-NTA coated foil [33].

### 2.4. Photocataytic Inactivation of Microorganisms

Conventional water disinfection methods can often lead to the formation of harmful by-products [127,128]. Disinfection using TiO_2_ as a photocatalyst has the power to inactivate microorganisms, as well as endotoxins released upon cell death [129]. Bacterial inactivation, using Pt/TiO_2_ as a photocatalyst, was initially reported by Matsunaga *et al.* in 1985 [130]. Since this discovery, a large body of research has been produced in an attempt to clarify the TiO_2_ photo-induced killing mechanisms. However, this research has centered on the use of TiO_2_ films and NP powders. While NPs provided more surface area for bacterial interaction, limitations in their use arose due to environmental issues from difficulties in filtration from solution and their inability to be effectively regenerated and reused. The use of anodized T-NTA eliminates these issues, as an array can easily be removed from a solution and regenerated without losing any structural or photocatalytic integrity.

Only recently has the inactivation of microorganisms using TiO_2_ nanotubes been reported [121,128,131]. Baram *et al.* reported on the inactivation of *Escherichia coli* (*E. coli*), examining many of the same parameters that affected organic mineralization [121]. Similar to organic decomposition, the application of an applied bias, formation of hydroxyl radicals, solution pH and the presence of inorganic ions, all affect the inactivation efficiency. Under UV irradiation (λ = 360 nm) and an applied bias of 1.5 V_SCE_, complete bacterial inactivation was achieved in 15 min (starting concentration of 10^6^–10^7^ cells/mL), whereas bacteria exposed only to UV irradiation, showed no decrease in concentration [121]. Acidic solutions (pH = 5) also showed an increase in inactivation as the negatively charge bacteria are attracted to the positively charged TiO_2_, increasing adsorption and cell death. Contrary to prior reports on TiO_2_ films and nanoparticles, TOC levels remained constant throughout the experiment; indicating that inactivated bacteria did not totally decompose into their final products (*i.e*., CO_2_).

Similar to organic degradation, coupling of T-TNAs with narrow bandgap semiconductor and metal composite NPs simultaneously enhanced the visible light activity and charge separation efficiency, increasing the bacterial inactivation efficiency. Inactivation of E. coli under visible light irradiation was achieved by Hou *et al.* through the use of Ag/AgBr/T-NTA [128]. This hybrid proved not only more effective than a Pt/CdS/T-NTA hybrid, but is also more cost effective and does not experience photocorrosion [131].

### 2.5. Carbon Dioxide Reduction

Increasing anthropogenic carbon dioxide emissions from mobile and stationary energy systems, as well as from various industrial processes have been speculated to play a role in global climate change [132,133,134,135] and have sparked multiple initiatives to reduce CO_2_ emissions. One method of CO_2_ mitigation is through post-treatment capture and utilization (recycle). Although a relatively inert molecule, there are numerous catalytic routes for converting CO_2_ to value-added chemical as outlined by Xiaoding and Moulijn [136]. Due to stability of the CO_2_ molecule, many of the catalytic conversion methods require substantial energy input, of which should be derived from a source(s) that do not contribute to further CO_2_ emissions. As a result, PEC and PC reduction process for the conversion CO_2_ on semiconductor electrodes has gained much attention in recent years. Studies have been extended to a variety of different avenues such as analysis of various semiconductor photocatalysts [137,138,139,140], modification of photocatalyst by depositing small amounts of metal [141,142,143] and metal complexes [144,145], and the effects of operating conditions [146,147,148]. In general, the PEC and PC reduction of CO_2_ to low-chain hydrocarbons and simple alcohols has not been examined extensively. Halmann showed the first report of the photoelectrochemical reduction of CO_2_ in 1978 [137]. The photoreduction was carried out using p-type gallium phosphate (p-GaP) as the photocathode with part or all of the energy supplied by light. The products were found to be formaldehyde (HCHO), formic acid (HCOOH), and methanol (CH_3_OH).

The majority of the studies on the PC reduction of CO_2_ have mainly focused around the use of nanoparticulate systems as reducing the particle size results in more active sites as well as simplified experimental setups. There are only a few reports on the PC reduction of CO_2_ using T-NTA in either gas or solution phase [143,149,150], which demonstrate μmol gram-catalyst^−1^ h^−1^ conversion rates. Products obtained from these studies consist predominantly of C_1_–C_2_ alcohols as well as C_1_–C_3_ hydrocarbons. Although the conversion rates are low, thermodynamic arguments are not sufficient to explain the limited conversion of CO_2_ to value added chemicals via a technical process. Reasons for such low conversions to value added chemicals are believed to be kinetic in origin. Since the σ-bonding orbitals of CO_2_ are deep down, it is deduced that only the π-orbitals of CO_2_ alone may be perturbed and an attack on the oxygen centers alone may be possible to activate CO_2_ [151]. As a result, a fundamental understanding of excited catalyst surface sites and surface states is necessary to engineer photocatalysts capable of effectively activating CO_2_ molecules [99,151,152] if a technical processes is to ever be developed.

## 3. Photoelectro-Catalystic Pollutant Degradation

The application of a low anodic bias, typically <1.5 V, to a TiO_2_ electrode can further enhance the photocatalytic (PC) degradation of pollutants [55,116,118,124,153,154,155,156,157,158,159,160,161,162,163]. In a photoelectrocatalytic (PEC) process, photogenerated charges are driven in opposing directions by the applied potential and subsequently hindering charge recombination. The forced bias also leaves photogenerated holes at the TiO_2_ surface, which can either interact directly with organic pollutants or react with H_2_O and OH^-^ to produce OH^•^ radicals. Bai *et al.* confirmed this improved efficiency through the comparison of typical methods used to decompose tetracycline [153]. The applied bias should be higher than the flat band potential of the TiO_2_, as well as high enough to fully mineralize the pollutant. Higher potentials generally perform better as they tend to fully degrade organics and the potentially harmful intermediate compounds [116]. The formation of intermediate compounds is also sometimes inhibited under a higher potential gradient as the generation and separation of the photogenerated charges is greater, causing the organic to degrade more completely and at a faster rate. Intermediate species are often difficult to fully degrade; analysis of the total organic carbon (TOC) should also be performed to unsure that full mineralization has occurred.

**Figure 8:**
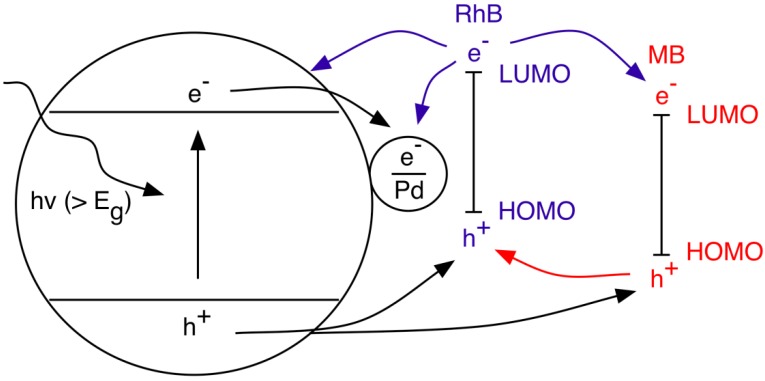
T-TNA assisted photo-decomposition of methylene blue (MB) and Rhodamine B (RhB). The HOMO-LUMO positions MB and RhB relative to the band positions of Pd/T-NTA provides a synergetic decomposition effect. Figure adapted from Reference [117].

Following on this line, an applied bias can also overcome decomposition retardation effects in solutions where multiple organic pollutants are present [117,154]. Zhang *et al.* reported the synergistic PEC decomposition of methylene blue (MB) and Rhodamine B (RhB) in the presence of Pd/T-NTA arrays [117]. The proposed mechanism for enhanced catalytic activity, illustrated in Figure 8, is based on the optimal band alignment between the highest occupied molecular orbitals (HOMO)-lowest unoccupied molecular levels (LUMO) levels of RhB and MB and the band edge positions of the N, F doped Pd/T-NTA arrays. In this scheme, photogenerated electrons in the LUMO of RhB can transfer to the Pd-NP, TiO_2_ CB or the LUMO of MB. This sensitization of the TiO_2_ expands the optical absorbance further into the visible light region, increasing the concentration of photogenerated holes that can be injected into the HOMO of both the RhB and MB. Synergistic effects are more significant for the RhB as holes from the MB can also be injected into the HOMO of the RhB.

## 4. Catalyst Modification

Titania is the most stable and widely used photocatalyst among all semiconductor materials investigated by far among those studied [164]. However, in terms of harvesting solar energy, the inherent large band gap of titania (~3 eV), limits the absorbance of light to wavelengths near the UV-spectrum (λ ≤ 390 nm) [165]. The UV-portion of the solar spectrum only constitutes ~5% of the total energy. As a result, utility of visible light (~40% of solar energy spectrum) photons is not realized. Therefore, extensive research efforts have been made to synthesize titania and titania composites compatible of harvesting visible photons. The visible light photoresponse of titania nanotubes can be greatly modified by various ways such as changing the shape and geometry of the nanotubes, controlling the crystallinity, doping, and coupling with other semiconductors, among others [12]. Through controlled doping with various metals, non-metals as well as semiconductors, a new class of materials has been developed with superior photocatalytic properties over plain T-NTA [12].

### 4.1. Metal Nanoparticles

Regardless of cost, the decoration of T-NTA with metal NPs is one of the most common methods used to increase the photodegradation efficiency of organic pollutants [117,155,166,167,168,169,170,171,172,173,174,175,176,177,178]. Metal NPs are typically chosen as it is easy to control the geometry and dispersion of these NPs onto the T-NTA and they are stable over a wide range of solution conditions. Choice of metal, size, and shape of the NP along with loading level are dependent upon which type of organic is being degraded and under what type of irradiation.

Nobel metal NPs (*i.e*., Au, Ag, Pt, Pd) are often chosen in photooxidation reactions due their suitable work functions. The Schottky potential barrier that exists at the TiO_2_-metal NP interface makes electron transfer from the TiO_2_ conduction band to the metal NP energetically favorable. This Schottky contact encourages rapid electron transport to the metal NP and facilitates in retarding recombination of photoinduced charges as the metal acts as an electron sink [179,180]. While Pt and Pd exhibit ohmic behavior and quickly discharge electrons to the surrounding medium, Au and Ag have a capacitive nature and tend to store charge [181,182]. When coupled with a semiconductor, Au and Ag can capture and store a fraction of photogenerated electrons produced by the semiconductor under irradiation, which in turn shifts the Fermi level to more negative potentials.

An increase in T-TNA photocatalytic performance has also been attributed to surface plasmon resonance (SPR) of deposited Ag nanoparticles [170,181,183]. Yu *et al.* [170] reported an increase in the mineralization efficiency of MO using T-TNA functionalized with 20 nm diameter silver/silver chloride (AgCl) NPs. Yu attributed this result to visible light induced surface plasmon resonance, suggesting that photogenerated electrons formed in the Ag NPs are injected into the conduction band of the TiO_2_, leading to the formation of oxidizing radicals. Simultaneously, holes are transferred to the AgCl, causing the oxidation of Cl^−^ ions to Cl^0^ atoms. In turn, the highly reactive Cl^0^ atoms are able to oxidize the organic pollutant and in the process become reduced once again to Cl^−^ ions. Alternatively, there is evidence to suggest that these observations could be the result of Ag electron storage instead of SPR [181,182]. Choi *et al.* [182] reported that when a metal is in contact with a semiconductor, photocatalytic enhancements are the result of the ability of the metal to capture electrons from the semiconductor and maintain a more negative Fermi level. However, particle size in an important factor in electron storage and the double-layer charging that stabilizes stored electrons is most effective when the particles are <10 nm [181,184]. The 6 nm nanoparticles examined in the charge storage studies [170,182] were well below the 20 nm particles used in Yu’s study.

Palladium (Pd) is frequently examined due to its relatively low cost compared to the other noble metals [117]. Mohaparta *et al.* [166] reported that palladium coated T-NTA, containing a loading level of 1.25 wt %, showed complete decomposition of MR in 150 min compared to only ~60% for the blank T-NTA [166]. However, above this loading level, the degradation of MR significantly decreased. It was suggested that the decrease in activity was the result of under utilized NPs deposited inside of the T-NTA. Loading the T-NTA surface with nickel (Ni) or iron (Fe) particles is a less expensive way to enhance the photocatalytic activity [175,185,186]. Nickel increases photooxidation in several ways: the amphiphilic behavior of Ni can lead to an increase in the adsorption of organic pollutant; the formation of redox-active p-type Ni(OH)_2_ in an aqueous solution leads to a p-n junction at the Ni/TiO_2_ interface, enhancing charge separation and radical formation [175].

### 4.2. Non-Metal Doping

Asahi and co-workers [187] performed pioneering work related to non-metal (C, N, F, O, S and P) doping in anatase TiO_2_ in regard to their capability to enhance photocatalytic properties of TiO_2_. Based on their theoretical calculation of the density of states (DOS), it was suggested that the most effective substitutional dopant appeared to be N, which induced maximum narrowing of the band gap of TiO_2_. Generally, when T-NTA is doped with nitrogen, N 2p states lie just above the O 2p states of T-NTA, thereby narrowing the band gap between. In addition, due to interstitial doping of N, there is also an intermediate state that forms in the middle of the band gap. In the case of carbon, sulfur and phosphorous doping, the states tend to form close to the valence band edge. Boron (B) forms an intermediate state near the conduction band edge, however. Moreover, Ti-substituted (Ti, subs.) nonmetals, sulfur and boron, also affect the band structure of TiO_2_, resulting in the formation of an intermediate state in the case of sulfur, and lowering of the CB minimum in the case of boron.

From the experimental perspective, N-doped T-NTA, investigated by Xu *et al.* [188], resulted in a substantial increase in photocatalytic response compared to undoped T-NTA. The N-doped T-NTA material was investigated for the degradation of textile dye Reactive Brilliant Red X-3B under visible light. Their study showed that 99% of the X-3B dye was decomposed by N doped T-NTA in 105 min, while with bare T-NTA, only about 59% degradation was achieved. Wu *et al.* [189] also carried out experiments to investigate N doped T-NTA for photocatalytic water-splitting. Under illumination of simulated solar light (AM 1.5, 100 mW cm^−2^), the N-doped T-NTA presented enhanced photoelectrochemical water-splitting performance. The enhanced water splitting property of the N doped T-NTA was attributed primarily to the expended optical absorbance behavior of the doped T-NTA in the visible light region. Similar investigation was also conducted by Sang and co-workers [190] where they synthesized both N doped T-NTA and C doped T-NTA composite materials for comparative study of their photocatalytic properties. The charge carrier density (N_A_) calculations suggested that C doped T-NTA demonstrated relatively higher N_A_ (5.31 × 10^20^ cm^−3^) than that of N doped T-NTA (N_A_ = 1.1 × 10^20^ cm^−3^). Further they also witnessed enhanced photoelectrochemical properties for hydrogen production from water with C doped T-NTA. However, there has been some disputes regarding the enhancement of photocatalytic properties of T-NTA after C-doping [12,191]. Nevertheless, enhancement in photocatalytic activities of C-doped T-NTA was observed by Park *et al.* [192]. In this study they prepared vertically grown carbon-doped TiO_2_ (TiO_2−*x*_C*_x_*) nanotube arrays with high aspect ratios were prepared via thermal treatment of clean T-NTA at elevated temperatures in a CO atmosphere. The synthesized TiO_2−*x*_C*_x_* nanotube arrays showed much higher photocurrent densities and more efficient water splitting under visible-light illumination (>420 nm) than pure TiO_2_ nanotube arrays. The total photocurrent was more than 20 times higher than that with a Degussa P25 nanoparticulate film under white-light illumination. Other studies also confirmed that C-doped T-NTA formed by thermal treatment in a carbon atmosphere increased the photoactivity [193,194]. Carbon doped T-NTA can also be synthesized by anodization in an organic electrolyte followed by thermal treatment in a reducing atmosphere [195]. Carbon or graphene has also been coated onto T-NTA to enhance PC [171,196,197]. A coating of partly graphitized carbon prior to Pt NP deposition was reported to increase the degradation of methanol by over 20 times [171]. Experimental data suggested that the increased conductivity facilitated charge transfer, and removal of intermediate and byproducts, during the photooxidative process. It was also reported that the carbon coating enhanced the Pt NP dispersion, which prevented the aggregation of NPs.

Phosphorus-doped titania nanotubes (P-T-NTAs) have been fabricated by anodization method [198] or by a wet chemical procedure with dimethyl phosphite as a precursor [199]. It was reported that P-doping shifts the band gap towards the visible light region [199]. When compared to pure T-NTAs, the optimal 0.75 wt % P-T-NTAs shows a band gap shift of 0.27 eV towards the visible light region. The photocatalytic activity of 0.75 wt % P-T-NTAs was tested using rhodamine B (RhB) as a model pollutant under a 9 W fluorescent lamp and was significantly better than the benchmark Degussa P25 nanoparticles due to the band gap narrowing and an increased surface area.

Generally, the edge of the valence band of T-NTA is at 3.0 eV *versus* NHE. The conduction band minimum is located at −0.2 eV *versus* NHE. Doping with S can perturb the CB and/or VB, and produces states in the band gap of T-NTA that absorb visible light. Umebayashi *et al.* [200] analyzed the band structures of S-doped TiO_2_ by *ab initio* calculation. According to their calculation, an electron-occupied level appears slightly above the VB, which contributes to visible absorption. The edge of the valence band is at roughly 2.0 eV *versus* NHE. Takeshita *et al.* [200] investigated the behavior of photogenerated charge carriers in S-doped T-NTA materials by transient absorption measurement in the region of mid-IR. The generation and activities of charge-hole photocarriers was found to be dependent on the S atom/particle size. However, they observed a much higher water oxidizing ability for the S-doped T-NTA material.

Yu *et al.* [201] studied the effect of fluorine-doping in titania nanotubes (F-T-NTA). It was observed that the F-T-NTA exhibited significantly enhanced photocatalytic efficiency compared with pure T-TNA. For example, methyl orange was degraded completely within 1.5 h with F-T-NTA, while only 47.4% of methyl orange was degraded using pure T-NTA [202]. The doped-F atoms can potentially promote the formation of oxygen vacancies in the T-NTA. The role of oxygen vacancies is to directly provide the formation sites of active species for photocatalytic reaction. The formation of O_2_^•−^ from chemisorbed oxygen or OH^•^ from adsorbed water requires the presence of surface oxygen vacancies.

In fact, it has also been pointed out that color centers, one type of intra-band gap energy state, are formed in F doped TiO_2_. The electron pair that remains trapped at the cavity gives rise to a F center; a positively charged F^+^ center is equivalent to a single electron residing at the oxygen vacancy. The electron-pair deficient oxygen vacancy is referred to as a doubly charged F^++^ center; the electrons left in the cavity can also react with adjacent Ti^4+^ ions to give Ti^3+^ centers. In most fluoride F-doped TiO_2_ photocatalysts, the main reason for the improved light absorption was the color centers formed upon F incorporation. For example, Li *et al.* [203] studied F-doped TiO_2_ from spray pyrolysis of H_2_TiF_6_ and found high photocatalytic activity in the decomposition of gaseous acetaldehyde under both UV and visible light irradiation.

Recently, iodine anion and cation doped T-NTA was synthesized by Su and co-workers [204]. Compared with an undoped sample, both cation and anion doped I-T-NTA show higher photocurrent and photocatalytic activity under UV-vis irradiation. First-principle calculations on undoped, iodine cation and anion-doped anatase T-NTA were performed and the results indicate that some new bands appear in the band gap and are beneficial for photogenerated carrier migration [204]. For cation-doped T-NTA, a strong interaction between the electrons in I 5p orbitals and in Ti 3d orbitals occurred and results in the CB shifting downwards. The band potentials of cation-doped T-NTA shift downwards to a larger extent and thus the VB has a stronger oxidative power than undoped and anion-doped T-NTA. Consequently, cation-doped TiO_2_ shows a higher photocatalytic activity.

### 4.3. Sensitization/Heterostructure

Coupling two or more semiconductors materials together is a common practice to achieve electrical or electrooptical properties otherwise not obtainable separately by each material. There are a variety of synthesis techniques to obtain such structures such as CVD, electrochemical, and wet-chemical. In many solar-based applications, large band gap materials (*i.e.*, TiO_2_) are sensitized with smaller band gap semiconductors that are often nanocrystal (NC) deposits or quantum dots (QD) [205,206]. The smaller band gap materials serve a similar purpose as dyes do in dye-sensitized solar cells (DSSC) in that they inject electrons excited via lower energy photons into the CB of the larger band gap semiconductor. A comparison of DSSC and QD solar cells is given by Hodes [207]. The advantage of using NC or QD as sensitizers lies within the quantum confinement effect as well the ability to tune the particle size [208] or composition [209] to absorb different wavelengths of light. Some semiconductor materials with narrow band gap include CdS [205,210,211], PbS [205,212], Bi_2_S_3_ [205,213], CdSe [214,215], CdTe [216,217] and InP [218], among others, have been investigated as sensitizers for T-NTA as an efficient absorber layer of the visible light spectrum.

A considerable amount of work has been dedicated to investigate CdS as sensitizer for T-NTA. In particular, the band gap of CdS (E*_g_* ≈ 2.4 eV) and its relatively high absorption coefficient in the visible region make it highly desirable for use in photovoltaics and photoelectrochemistry in comparison with other semiconductors. A coupled CdS/TNA photoanode may be suitable for efficient solar energy conversion. In a study by Banerjee *et al.* [219], T-NTA were filled with CdS nanoparticles (size range 70 to 140 nm) by electrochemical deposition and have witnessed a 8–9 fold increase in photoactivity compared to that of pure T-NTA. They suggested that the absorption of visible light component (λ ≥ 420 nm) was entirely due to the presence of the CdS nanoparticles, which constituted about 68% of the total photocurrent generated. Smith and Subramanian [37] devised a flexible photoanode material by combining anodically growing T-NTA on a titanium mesh with a TiO_2_ nanoparticle overlayer and in subsequent steps, the TiO_2_ nanotube/nanoparticle heterostructure was further sensitized with CdS nanocrystals by the SILAR process. The highest photocurrent conversion efficiency (35%) was obtained by sensitizing the T-NTA-TiO_2_ nanoparticle composite with CdS nanocrystals. In a different but relatively recent study [220], CdS-modified short, robust, and highly-ordered T-NTA composite array was developed using sonoelectrochemical anodization and sonoelectrochemical deposition method. The composite array demonstrated highly efficient visible-light hydrogen generation properties with remarkably stable photoelectrocatalytic properties. Photocurrent response obtained using with the CdS/T-NTA electrode was 7 times higher in comparison with the bare T-NTA electrode. Despite the widespread use of CdS as a sensitizer, it is prone to corrosion by hole accumulation under irradiation without a proper redox couple. Wilson *et al.* [221] reported the addition of Na_2_S to the electrolyte solution during the photodegradation of MO using a CdS-T-NTA array. Na_2_S was added as the sulfide anions can assist in the scavenging of holes in the VB of CdS, thus stabilizing the CdS nanocrystals. Concentrations of Na_2_S, limited to 0.02 M, also improved MO degradation through enhanced separation of photogenerated charges.

The use of CdSe as a sensitizer has shown higher performance over CdS due to its lower band gap. Hossain and co-workers [222] investigated T-NTA-CdSe nanocomposite based material by decorating the nanotubes with bubble like CdSe nanoclusters by chemical bath deposition technique. Other methods to simultaneously synthesize and attach CdSe onto T-NTA include the common successive ionic layer absorption and reaction (SILAR) method [223] as well a solvothermal deposition [224]. Coupling CdS with CdSe in tandem on T-NTA has also been investigated [225] and showed the CdS/CdSe couple had the highest performance over CdS or CdSe alone on T-NTA.

#### Metal Oxides

Sensitization is commonly carried out with semiconductor sulfides or selenides as these materials can utilize visible and near-IR photons and can easily be synthesized to dimensions less than their Bohr radius. Use of nanodimensional metal oxides to enhance the photoactivity of T-NTA is another method. Although metal oxides generally have larger band gaps, they are generally more stable. Additionally integration of different metal oxides and metal oxide based semiconductor materials into T-NT has resulted in the improvement of the photocatalytic properties of the composite system [226]. The incorporation of such oxides in to T-NT is to alleviate/modify the charge carrier recombination behavior in individual oxides and that in turn changes the recombination properties of the composite. The unidirectional transfer of photogenerated electron and holes can be achieved by suitable matching of the CB and VB bands of the oxide and the T-NT. However, the effective conduction of the photogenerated charge carriers and reduced recombination losses depends on various properties exhibited by the oxide such as surface area, defect density, crystallinity, particle size-shape effect and quantum size effects. For example, CdO is an n-type semiconductor with a band gap of 2.32 eV and its conduction band is ~1.1 eV more negative to that of T-NTA. Therefore, coupling of CdO with T-NTA would not only help harvesting the visible portion of the sun light but also helps in effective charge separation. Recently, the current authors conducted a study to evaluate the PEC properties of T-NTA/CdO composite material [227]. The photocurrent response of the composite materials achieved in this study was about two times higher than that of bare T-NTA. It was asserted that the electronic properties, in terms of charge separation, was much improved in the case of the T-NTA/CdO composite because of the presence of small band gap CdO semiconductor material. The charge carrier density (N_A_) calculations, using Mott-Schottky analysis under illumination of the T-NTA and the T-NTA/CdO composite materials indicated values 3.27 × 10^17^ cm^−3^ and 1.52 × 10^18^ cm^−3^, respectively. The increased N_A_ for the composite material is possibly because of effective separation of photo-generated electron and holes compared to that of pure T-NTA due to the formation of a localized electric field. The separation of the electrons and holes increases the lifetime of the charge carriers and in turn increase N_A_. Incorporation of CdO with T-NTA causes easy transfer of photo-generated electrons from the conduction band of CdO to that of conduction band of TiO_2_. 

Coupling titania with WO_3_ has been found in many studies to enhance the photocatalytic activity. This is because of the narrow band gap energy (2.6 eV) exhibited by WO_3_ making it easily excitable by visible light. Therefore, more visible light can be harnessed by WO_3_ from the sunlight spectrum. Another virtue of WO_3_ is its remarkable photostability in acidic aqueous solutions, which makes it attractive in applications such as the treatment of wastewater contaminated by organic acids. Earlier studies of the photoelectrochemical behavior of both polycrystalline and monocrystalline WO_3_ provided instructive knowledge for the development of photocatalytic water-splitting systems. In fact, enhanced photocatalytic properties have been observed by coupling WO_3_ into T-NTA. For example, in regard to enhanced photo-water splitting properties, Smith and co-workers [228] demonstrated a novel process to fabricated a nanotubular composite of T-NTA–WO_3_ via anodic oxidation of titanium in a single-step process using phosphotungstic acid as the tungsten source. The composite material demonstrated an increase in conversion efficiency in PEC water splitting by incorporating WO_3_ compared to T-NTA prepared under similar conditions. Similar observations were also reported by Lai *et al.* [229], where they fabricated WO_3_–T-NTA nanotubes by wet impregnation method. The composite material exhibited better photoelectrochemical water-splitting characteristics under visible illumination. Compared to that of the bare T-NTA, a three times increase in photocurrent density and efficiency was achieved with WO_3_–T-NTA composite nanotubes. The authors explained their observations with increased charge carrier separation, minimized recombination losses and enhanced transportation of photo-induced holes in the composite nanotube structure. It should be noted, however, the CB minimum for WO_3_ is lower than that of titania. As a result, the charge transfer mechanism is slightly different and may follow a Poole-Frenkel type mechanism [228].

Similar photocatalytic properties can be expected by coupling iron oxide (Fe_2_O_3_) with titania as the band gap energy for iron oxide is ~2.1 eV [230]. In absence of electron or hole annihilators, the recombination of the photo generated charge carriers is found to be very rapid (within 1 ns) when irradiated with optical illumination. However, in the presence of annihilators, the electrons and holes can function as moderate reductant and powerful oxidants, respectively. For example, S^4+^ species, such as aqueous SO_2_ *etc*., have shown to be readily oxidized on the surface of α-Fe_2_O_3_. Using Fe^3+^ as electron scavengers, α-Fe_2_O_3_ is capable of oxidizing H_2_O to evolve oxygen gas. A bias potential can also help to achieve total H_2_O splitting under visible irradiation. A number of organic compounds (e.g., salicylic acid,) can also be photocatalytically degraded on α-Fe_2_O_3_ photocatalysts, either by adding sacrificial scavengers or through the application of a bias potential, although the efficiency remains to be improved.

**Figure 9:**
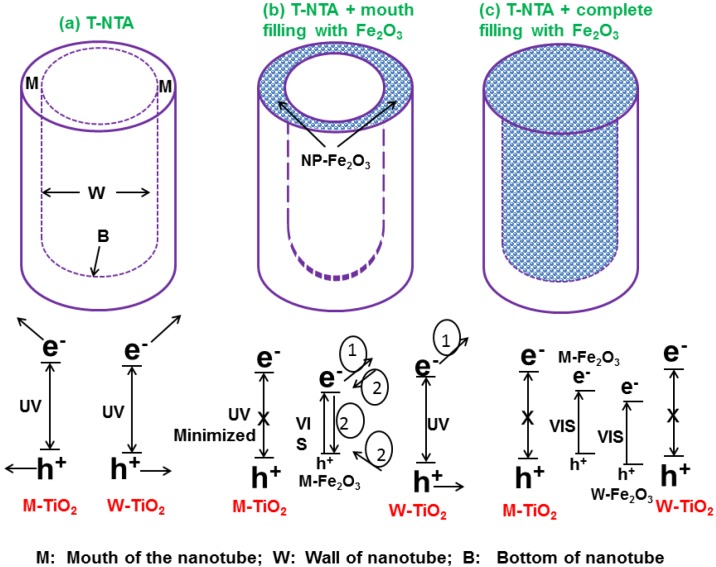
Schematic of Fe_2_O_3_ decoration on T-NTA through electrodeposition and proposed charge transfer mechanism for Fe_2_O_3_ decorated. Adapted from Reference [230].

**Figure 10:**
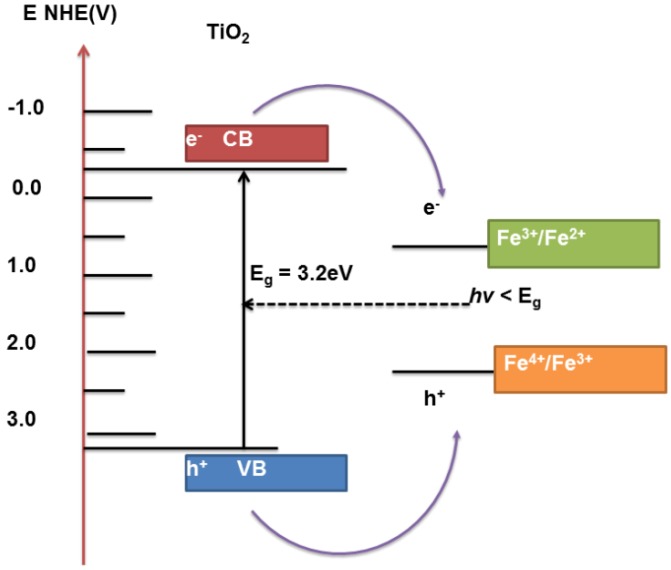
Schematic of charge transfer from excited T-NTA to different bands of Fe-ions. Figure adapted from Reference [232].

Muramatsu *et al.* [176] modified the surface of T-NTA with iron oxide particles at a molecular scale by the chemisorption–calcination cycle method. The iron oxide-surface medication endows T-NTA with a high level of visible-light-activity, along with the increased UV-light-activity. The iron oxide-surface sensitized T-NTA exhibits a UV-light activity even higher than that of highly active TiO_2_ particles. Using a simple electrodeposition technique, Cong and co-workers [185] synthesized α-Fe_2_O_3_ dispersed T-NTA heterostructure for the degradation of phenol. The hetero-nanotube composite was more effective in the photoelectro-Fenton degradation of phenol than that of α-Fe_2_O_3_ nanoparticles or bare T-NTA. The composite α-Fe_2_O_3_/TiO_2_ electrodes showed an enhanced absorbance in visible light region and had good stability to photoelectro-Fenton reactions. Similar observations were made by Mohapatra *et al.* [231] by filling T-NTA with Fe particles via pulse electrodeposition. Subsequent heat treatment formed the α-Fe_2_O_3_/T-NTA electrodes. More recently, Jeon *et al.* [230] carried out both theoretical and experimental investigation of the effect of nanocrystalline hematite particles (α-Fe_2_O_3_) decoration on to the T-NTA arrays and their photocatalytic properties as a composite material. Deposition of α-Fe_2_O_3_ was performed in three ways (Figure 9): (i) deposition such that the NP would decorate only the mouth surface of T-NTA; (ii) complete filling of the inside of T-NTA; (iii) and an even full-covering of the T-NTA top surface as shown schematically in Figure 10. The PEC study was carried out with AM 1.5 irradiation and demonstrated a substantial decrease in the PEC activity when hematite covered the full surface of T-NTA and loaded on the mouth surface of T-NTA. This is rather usual as an enhanced photochemical response is expected with the addition of α-Fe_2_O_3_ on T-NTA. It is to be noted that the distribution characteristics of electrodeposits in to T-NTA substrate can potentially alter some of the important properties of T-NTA such as catalytic activity [227]. In this case, the authors explained the unusual results based on the photogenerated charge transfer mechanism as achieved with various distribution scenarios in the composite materials as shown in Figure 10.

For the case of bare T-NTA (case I, Figure 9) the UV fraction of light absorbed by T-NTA excites not only the mouth surface nanotubes but also the inner wall surface of the T-NTA. Therefore, in the most simplistic form, when UV is irradiated, both surfaces are excited, and the subsequent generation of electron and hole pairs contributes to photocurrent generation (case a). However, when α-Fe_2_O_3_ particles are present on the mouth of T-NTA, as shown in case b, the composite absorbs the UV as well as visible portion of the energy, minimizing the effect of the mouth surface T-NTA in contributing to the overall photocurrent generation. The author argued that the significant reduction in photoactivity of the surface covered α-Fe_2_O_3_/T-NTA hybrid architecture (case II) might be because of rapid transfer of CB electrons and VB holes of T-NTA to CB and VB of hematite phase, respectively. The subsequent charge annihilation may occur through the pathway 2 of case b. In the case of α-Fe_2_O_3_/T-NTA composite completely covered by the α-Fe_2_O_3_ (case c) most irradiated UV photons are absorbed by the hematite phase; thereby no activation of T-NTA would be possible by the UV light. Experimentally it was found that the overall photocurrent response for case c is more than case b while lesser than case a, possibly because of the following reasons: First, the enhancement is due to large amount of surface hematite phase in case c than case b and thereby more photons are absorbed by hematite, contributing to the enhanced photocurrent. Second, if the inter wall TiO_2_ has much lower photoactivity, the relatively inactive inter wall surface becomes active by the filled hematite. Finally, if the inter wall TiO_2_ is as active as the mouth surface TiO_2_, the recombination occurring at hematite and TiO_2_ interface (hematite/TiO_2_ contact) is minimized or vanishes due to replacement of the inter wall TiO_2_ with the hematite (hematite/ hematite contact).

Iron oxide (Fe_2_O_3_) NPs have also been used in Fenton-related processes, where Fe_2_O_3_ is used to catalyze reactions with hydrogen peroxide for contaminant oxidation. T-NTA electrodes participate in the electro- or photoelectro-versions of this process, as they generate additional hydrogen peroxide through the electrochemical reduction of oxygen [185]. It was reported that both UV and visible light photoelectro-Fenton process provided better removal of phenol than electro-Fenton with an applied bias. Loading levels were also optimized in this experiment as not to clog the T-NTA, as well as limit the transport of photogenerated holes from the VB of TiO_2_ to the VB of the Fe_2_O_3_, potentially causing e-h recombination.

Other metal oxides composite materials, such as NiO/T-NTA, SnO_2_/T-NTA have also been investigated for their photocatalytic properties [233,234]. The incorporation of NiO in to T-NTA resulted in remarkable enhancement in photoactivity under visible light [233]. In fact, the photocatalytic properties of NiO/T-NTA were comparable to those obtained with N-doped T-NTA. Limited research efforts have also been made to investigate SnO_2_/TiO_2_ composites for their photocatalystic properties [234]. The enhanced photoactivity exhibited by SnO_2_/TiO_2_ composite materials was partly because of better charge separation in the composite material. It is well known that the proper placement of the band energies of the individual semi-conductor is essential for the charge separation to ensure high photocatalytic activity due to fast electron transfer from TiO_2_ to SnO_2_. The band gap energy difference between SnO_2_ and TiO_2_ (3.8 and 3.2 eV, respectively) makes the former as an electron sink for photogenerated electrons. Consistent with this theoretical understanding, Hou *et al.* [234] also found that SnO_2_/T-NTA composite structure exhibited remarkable photocatalytic properties as efficient absorber for methylene blue (MB) due to decreased electron-hole recombination behavior of the composite. Recently, Li *et al.* reported that the strong dielectric and ferroelectroic properties of BaTiO_3_ could improve the photooxidation of methylene blue (~10% over blank T-NTA) [235]. The improvement was attributed to both the greater spatial separation of the photogenerated charges and its band position relative to TiO_2_. Both the CB and VB of BaTiO_3_ are located at lower potentials those of the TiO_2_, making electron transfer to the CB and hole transfer from the VB of the TiO_2_ thermodynamically favorable.

A significant red-shift in the optical properties was reported for transition metal (Fe, Cr, V, Mn, and Ni) doped TiO_2_ composite materials based on the work of Umebayashi and co-workers [236]. Generally upon doping with transition metals (Fe, Cr, *etc*.), the dopant ions can occupy two different positions in a semiconductor T-NT lattice, namely the substitutional and/or the interstitial vacancies. This occupancy primarily depends on the ionic size of the dopant. Dopants of size similar to that of the matrix cation tends to occupy the substitutional sites while the smaller sized dopant prefers the interstitial positions. Doping of transition metals in to a wide band gap semiconductor photocatalysts such as T-NT causes changes in two specific properties of the photocatalyst: the bulk electronic structure and the surface properties. The change in the electronic structure is brought about by the changes in the position of the Fermi level. On the other hand, the variations in surface property of the T-NT is related to the thickness of the space charge layer, changes in the concentration of surface states, and the initiation of photo-corrosion processes [226]. When a transition metal (M) is doped into the T-NT, the cationic species of the dopant (M*^n^*^+^) are incorporated in the substrate through the substitution of the Ti^4+^ cations. This substitution causes the formation of oxygen vacancies to maintain the electronic neutrality when the dopant cation is trivalent (*ex*. Fe^3+^, Cr^3+^) by the following reaction [226]:

M_2_O_3_ → 2|M^III^|_Ti′_ + V_o_^••^ + 3O_o_ (25)

where, M is the transition metal; V_o_^••^ is the oxygen vacancy and O_o_ is the oxygen atom at its normal lattice site. Therefore, some of the dopant centers will have oxygen vacancies as its nearest neighbor. Similarly, for a pentavalent transition metal ion such as V^5+^, the reaction in Equation 25 can be written as [226]:

2M_2_O_5_ → 4|M|_Ti_^5^^•^ + 5V_O_^4^′ + 10 O_o_ (26)

where 5V_O_^4′^ represent the cation vacancy and M|_Ti_^5^^•^ represents the defect site that can act as good electron acceptor. In fact, intra-band gap energy levels can form by conversion of V^5+^ to V^4+^ and V^3+^ by the acceptance of one or more photoelectron. Creation of such intra-band gap was suggested to cause a significant amount of red-shift in optical absorbance. In fact, enhanced photoactivity has also been observed upon doping of transition metals into T-NTA [237]. Ouyang *et al.* [238] introduced Fe in to T-NTA and their composite material demonstrated much higher visible-light photocatalytic activity for the degradation of MB than bare T-NTA. They suggested that Fe incorporation effectively promoted the separation and diffusion of photogenerated charge carriers, which is responsible for the enhanced photocatalytic activity. In a different effort [239] the photocatalytic activity of Cr doped T-NTA was evaluated in terms of degradation of phenol and photoreduction of CO_2_ into methanol and ethanol under UV and IR irradiation. They found that Cr-doped T-NTA exhibited much higher photocatalytic activity than that of bare T-NTA.

Further modification of doping elements was carried out in terms of doping T-NTA with transition metal ions [236]. Various ions such as Fe^3+^, Cr^3+^, and V^5+^ have been used to dope with TiO_2_ and the composite materials formed were evaluated as photocatalysts. Sun *et al.* [232] incorporated Fe^3+^ ions into T-NTA by a single step fabrication process. The enhanced photoactivity of the composite in relation to the removal of MB aqueous solution was attributed to the presence of Fe^3+^ in the T-NTA. The authors suggested that optimum photocatalytic activity could be achieved upon doping at a relatively weak level of Fe^3+^ ions. The effect of optimum Fe^3+^ doped in TiO_2_ can be explained from the standpoint of efficient separation of photogenerated electron–hole system. Figure 10 shows a schematic of the energy diagram for a Fe^3+^–T-NTA system. Under UV irradiation, Fe^3+^–T-NTA composite in MB solution undergoes following reactions:

TiO_2_ + hν → e^−^_CB_ + h^+^_VB_ (27)

Fe^3+^ + h^+^_VB_ → Fe^4+^ (28)

Fe^4+^ + OH^−^_ads_ → Fe^3+^ + OH*_ads_ (29)

Fe^3+^ + e^−^_CB_→ Fe^2+^ (30)

Fe^2+^ + O_2(ads)_ → Fe^3+^ + O^−^_ads_ (31)

The photogenerated hole can be trapped by the Fe^3+^ ionic species (Equation 28) due to the energy levels for Fe^4+^/Fe^3+^ above the VB edge of anatase T-NTA. Subsequently migration of the trapped holes in Fe^4+^ to the surface results in the absorption of hydroxyl ion to produce hydroxyl radical (Equation 29). In the same way, Fe^3+^ also traps photogenerated electrons as described by Equation 30 due to the energy level for Fe^3+^/Fe^2+^ (0.771 V *versus* NHE) below the CB edge of T-NTA. Subsequently, Fe^2+^ could be oxidized to Fe^3+^ by transferring electrons to absorbed O_2_ on the surface of T-NTA (Equation 31). In addition, among the three different chemical states of Fe ion, Fe^3+^ is relatively stable due to its semi-full 3d electronic configuration (3d^5^) and the charge trapped by Fe^4+^ or Fe^2+^ can easily release to return back to Fe^3+^ and then participate in photocatalytic reaction. These factors are apt to inhibit the recombination of photogenerated carriers and improve the photocatalytic activity of photocatalysts. However, when Fe^3+^-doping amount exceeds a certain level, due to the decrease of the distance between trapping sites, Fe^3+^ may also act as a recombination center of the photogenerated electrons and holes according to Equation 30.

## 5. Theory

The main scope of this review thus far has been focused on metal oxide nanotube arrays and their photocatalytic and photoelectrochemical applications, particularly titania nanotubular arrays. All photo/electro-catalytic reactions are a surface phenomenon and are driven by favorable surface/absorbate energetics. The use of computational models can give valuable insight into the interaction and energetics of surfaces and absorbing/desorbing species to engineer better performing catalysts. Several studies are available in the literature that have focused on the theoretical investigation of metal oxide nanostructures and the examination of their different properties in various applications ranging from photocatalytic and photovoltaic to biosensing [240,241,242,243,244] (and references there within).

This section of the review will examine a broader perspective and briefly overview the theoretical investigations on the various applications of TiO_2_ nano-structures ranging from catalysis and surface chemistry, to solar-based applications such as dye-sensitized solar cells (DSSC). The electronic structure of the metal oxide surfaces and the surface adsorbants determine the properties and its application. Various theoretical methodologies adapted for investigating the properties related to catalytic and photocatalytic applications will also be covered.

### 5.1. Computational Protocol

In general, the realistic description of metal-oxide surfaces is provided by the periodic slab model [245]. The slab model defines a periodic unit cell exhibiting the crystal surface of interest and the area of the unit cell of the slab can be made as large as necessary to represent a given coverage of adsorbates. The number of atomic layers in the slab can be determined by calculating the properties of interest as a function of the slab thickness until convergence is achieved. In general, a constrained slab model is used, where the atomic position of the atoms in the bottom layers is kept fixed at the bulk value, whereas those of the uppermost layers are fully relaxed. In principle, these slab models use plane waves as the basis set, and since these functions are intrinsically periodic, it is necessary to replicate the slab model in the third direction as well as leave a sufficiently large vacuum region between the interleaved slabs. Similar to the slab thickness, the vacuum width needs to be controlled in the model since the computational cost increases with the unit cell size. Presently, there is a number of efficient and parallel computer codes available such as VASP [246], CASTEP [247], ABINIT [248], Quantum Espresso [249], CRYSTAL [250], and SIESTA [251] to mention a few.

Modeling the main steps involved in a DSSC requires one to model electron transfer phenomena, which can be properly described by a quantum chemical method [243]. Nowadays, density functional theory (DFT) is probably the most widely used method in computational chemistry, yielding an accurate prediction of several ground and excited state properties [252]. A wide variety of complex and accurate exchange–correlation functionals have been recently developed that permit more accurate computation, especially the hybrid functionals, which explicitly include a fraction of Hartree–Fock (H–F) exchange such as B3LYP [253] or PBE0 [254], which are most popular for molecular systems. However, for extended solid systems, the hybrid functionals concerns band gaps to some extent. Hybrid functionals are still not largely used for solids, as the evaluation of the exact H-F exchange contribution at long range is computationally demanding when plane waves are considered as basis sets [255]. When localized (Gaussian) basis sets are chosen however, hybrid functionals can be efficiently applied to periodic systems [245]. The global hybrids (GH) such as PBE0 [252] are proven to yield impressive performances for studying the band gap of semiconducting systems and excited state properties of molecular systems at time-dependent DFT level. In certain cases however, a correction of the exchange potential form to obtain the correct long-range behavior is required and obtained through the use of range-separated hybrids (RSH). These functionals [256] have shown promising results particularly dealing with through-space charge-transfer transitions, for which standard GH fail to accurately reproduce the experimental data [252]. However, the latest generation functionals, such as M06-2X and M08-HX, actually perform better than the RSH (CAM-B3LYP) for describing charge transfer excitations with intermediate spatial overlap [256]. Overall, PBE0 appears as a good compromise for a consistent description of both ground and excited state properties of molecules, surfaces, and solids, as needed to model DSSCs, without introducing any empirical and material-dependent parameters. In order to tune the band gap, a commonly used approach is adapted, *i.e*., to add two parameters to the local density approximations (LDA) or Generalized gradient approximation (GGA) exchange-correlation functional. The resulting method is often referred to as DFT + U, or more specifically, as LDA + U or GGA + U. One advantage of LDA + U or GGA + U over the hybrid functionals described above is that the additional computational cost is minimal, although it has the problem that the U parameter empirically determined is usually effective only on one energy band (3d, 4d, or 5d in the case of transition-metal oxides), whereas the introduction of a part of the Fock exchange modifies the whole electron density of the system. Apart from the band structure calculation, a growing interest is nowadays dedicated to the so-called GW quasi-particle approach [257]. The quasi-particle approach permits simulation of the ejection or the absorption of an electron caused by an incident photon, *i.e*., the transition from an N-electron state to an N ± 1 state. This approach, in principle allows for a direct comparison of the calculated results with photoemission or inverse-photoemission experiments. Environmental effects on the electronic properties should be taken into account, however. Solvation in case of DSSCs can be routinely taken into account by considering implicit solvation models such as polarizable continuum models (PCM) [258]. In PCM model, the solvent is modeled by a (continuum) medium characterized by a static dielectric constant, where the polarizibility and polarizable property of the solute is considered. Implicit and/or explicit models can also address the bonding interactions between the solute and the solvent. Explicit inclusion of the first solvation shell in the electronic calculation and embedding the resulting cluster in a PCM to simulate the bulk solvent effects is a preferred choice. Surface interactions could also significantly tune the spectral properties of dyes. A powerful tool to include such effects combines the ONIOM method [259] with electronic embedding (EE) potentials.

The wave function of the chemically relevant part of the system can be polarized by the electrostatic contribution of its surroundings, by an appropriate division of all subparts of the system. The most accurate way to approach electronic excitations in the framework of DFT is its time-dependent implementation, usually called TD-DFT, based on the Runge−Gross theorems [260], which may be seen as a consequence of the time dependent Schrödinger equation. TD-DFT thus extends the standard formulation of DFT described above to time dependent phenomena. Interfacial electron transfer is the primary step in many solar energy conversion devices, since it creates free charge carriers upon the absorption of a photon. TD-DFT is based on the fact that the linear response function—that is, how the electron density changes when the external potential changes—has poles at the exact excitation energies of a system. Hence, for fast electron transfer processes in photovoltics and solar energy production this methodology has proven to be an appropriate method. In the case of photovoltics, theoretical modeling and understanding of interfaces composed of two qualitatively different species (metal-oxide surface and organic moiety) is challenging and intellectually stimulating. There are two different strategies for modeling the interfacial electron transfer in real time: (i) fully quantum-mechanical electronic and vibrational dynamics are investigated using a simplified model of the interface [261,262]; and (ii) an explicit atomistic representation of the interface is combined with quantum-classical or semi classical electron-vibrational dynamics [263,264,265]. Both strategies have their advantages: simplified models allow the investigators to systematically vary the model parameters and study the influence of various interfacial properties on the electron dynamics. Atomistic simulations, on the other hand, can treat a much more realistic interface, including the time-dependent geometric and electronic structure of the chromophore, the surface, and the chromophore surface binding. Additionally, *ab initio* time-domain atomistic simulations have been performed and investigated the rates and mechanisms of electron injection [243,264,266].

#### 5.1.1. Clean Titania Surface

**Figure 11:**
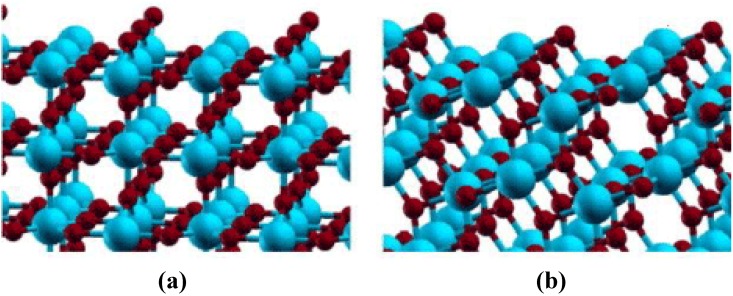
(**a**) Rutile (110) surface; and (**b**) Anatase (101) surface of titanium dioxide (Adapted from [270]).

Titanium dioxide is a transition metal oxide crystallizing in three polymorphs in nature: rutile, anatase, and brookite. The bulk and surface properties of rutile and anatase have been extensively exploited in last four decades. Due to its readily availability, the rutile polymorph has been extensively studied compared to the other two phases [242]. The stable surfaces of rutile polymorph (110) (100) and (001) have been the most investigated. The 1 × 1 termination of (110) rutile surface is the most stable prototype titania surface, which has been the point of interest for fundamental research. However, the anatase form attains industrial importance since titania nanomaterials adopt this phase at low to moderate temperatures due to its low surface energy [267]. The (101) and (100) surfaces of anatase polymorph has demonstrated application in solar cells due to their high photocatalytic ability. A significant difference between the electronic properties, band gap, adsorption properties of above two surfaces of anatase has been studied [75,268]. The computational modeling of titania structures has been done using H-F as well as DFT utilizing different basis functions such as: (i) all-electron calculations-LCAO approach; (ii) pseudopotential calculations with localized basis sets and plane wave basis sets. To model bulk rutile and anatase surfaces, a conventional unit cell of 6 atoms for rutile and 12 atoms for anatase were considered [269]. Consequently, rutile (110) layer as a trilayer consisting of two in-plane Ti, two in plane O atoms, and two bridging O atoms above and below the central Ti-O layer. Likewise, anatase (101) layer contains two Ti and four O atoms in its primitive cell [269]. A depiction of rutile and anatase unit cells is given in Figure 11.

**Figure 12:**
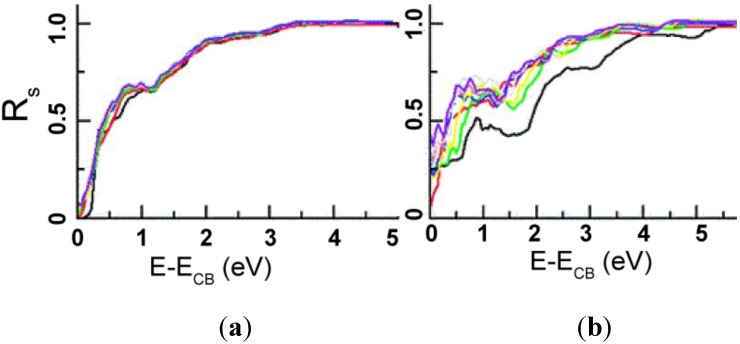
Rs(E) against E for slabs computed using the B3LYP functional with CRYSTAL06 for anatase (101) (**a**) and rutile (110) (**b**) for slabs 3 (black), 4 (red), 5 (green), 6 (blue), 7 (yellow), 8 (brown), 9 (gray), and 10 (violet) layers thick. (Figure adapted from Reference [274]).

Recent theoretical studies using *ab initio* H-F and DFT shows the most stable surfaces of anatase and rutile polymorphs are (101) and (110), respectively [271,272]. Using five different hamiltonians (HF, LDA, PBE, B3LYP, PBE0) and two different type of basis sets (LCAO and PAW) various properties of rutile and anatase titania has been studied by Labat *et al.* [272]. The best description for all properties has been obtained using hybrid PBE0 level. They also confirm the stability of anatase phase compared to rutile [272]. A careful analysis of the band structures for both the phases of titania confers that the valence band correspond mainly to the O 2p states, whereas the conduction band comprises of Ti 3d states. The band gap calculated for both rutile and anatase phase largely depends on the exchange-correlation functionals and number of layers in the slab [269]. The oscillations in the electronic structure and structural properties of rutile (110) slabs are due to the change in the hybridization of the O 2p and Ti 3d states. Hence, the surface energy values show a pertinent oscillation between the odd and even layer slabs. Subsequently, there is no agreement in the literature for TiO_2_ surface energy values: localized atomic orbitals have report values in the range of 0.42–0.92 eV for rutile (110) [272]. The calculated surface energy values largely depend on the method of computation used. However, the band gap converges monotonically for anatase (101) on the bulk value. Concomitantly, the surface energy for this surface converges monotonically, irrespective of the even or odd layered slabs [269]. The HF exchange determines the accuracy in predicting the band gaps in such structures. Hybrid functionals like B3LYP is found to be more comparable to the experimental data [273]. The computed band gaps have large effect on the structure irrespective of the Hamiltonian or basis set used for calculation. In order to determine the appropriate slab thickness in predicting the conduction band local density of states of a true surface, a comparison of ratio of states per atom on the surface of atoms and on the other atoms in the slab Rs(E) (ratio of states per atom on the surface atoms and on other atoms in the slab) was calculated. From the plot of Rs(E) *vs*. energy, it is determined that three layer anatase slab gives an appropriate model of the surface (Figure 12) [274]. For rutile the convergence is less rapid due to oscillation as discussed before. Often constrained slab models, where atoms in one or more bottom layers of the slab are not allowed to move, are used for studying surface phenomena. There is a slight change (0.4 to 0.8 eV) in band gap observed for the anatase and rutile phase with constrained slab model [274]. This is attributed to the shift in valence band maximum in constrained slabs, which in principle should not affect the adsorption and electron transfer into the top layer.

#### 5.1.2. Photocatalysis

A number of theoretical models have been developed to study the movement of charge in molecules and solid-state structures [243]. The interaction of the discrete, localized orbitals of nonmetallic compounds and continuous bands of delocalized quantum states create certain challenge in studying the interfacial region as well as the electron transfer from interfacial to bulk material. This electron transfer process is the major research focus in different photocatalysis for environmental remediation, photoelectrolysis, and solar energy conversion and storage [240,264]. New designs and fabrication techniques have been investigated especially for DSSC [240,275,276].

**Figure 13:**
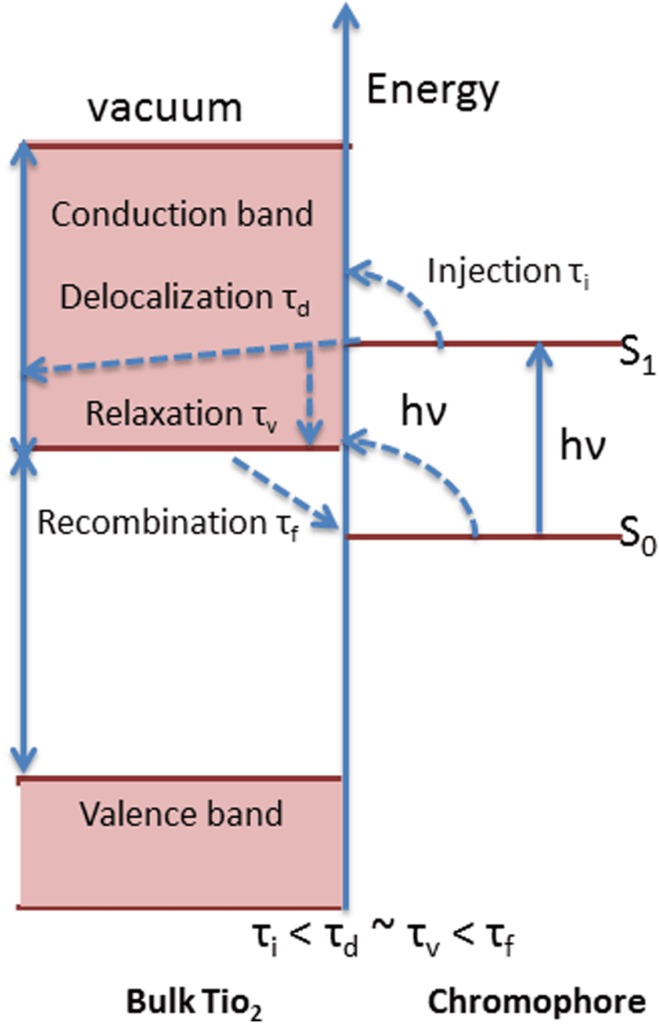
Energy diagram of the chromophore-TiO_2_ interface. An absorbed photon promotes an electron from the ground state (S0) of the dye located in the semiconductor energy gap into an excited state (S1) that is in resonance with the conduction band (CB). Typically, the dye-excited state is well inside the CB. An additional direct photoexcitation from the dye ground state into semiconductor state near the CB edge becomes possible with a strong coupling as in the catechol-TiO_2_ system. In some systems, such as alizarin-sensitized TiO_2_, the dye-excited state is located near the edge of the TiO_2_ CB. Efficient electron injection into the edge of the CB avoids the energy and voltage loss by relaxation to the CB edge that is inevitable if injection occurs deep into the CB. The injected electron delocalizes from surface to bulk, simultaneously relaxing to the bottom of the CB owing to coupling to vibrations. If the electron returns to or remains trapped at the surface, it recombines with the positive charge residing on either the chromophore or the electrolyte mediator. (Adapted from Reference [243]).

The general mechanistic stages (Figure 13) of interfacial photochemical processes/reactions are worth considering as they occur in gas/solid and liquid/solid heterogeneous systems. The driving force of such processes/reactions is absorption of the light free energy resulting in the intrinsic (band-to-band) or extrinsic (ionization of defects) excitation of the solid and the photoexcitation of surface states, which leads to the photogeneration of charge carriers in the catalyst, namely electrons (*e*^−^) and holes (*h*^+^) in the conduction and valence bands, respectively. As the positions of the energy levels of defects and surface states are within the forbidden energy gap (bandgap), photoexcitation (photoionization) of the defects and/or photoexcitation of the surface require photons with less energy compared to band-to-band photoexcitation. Recombination and carrier trapping events of excitation decay restore the initial state of the solid. Concomitantly, charge carriers can also be partly trapped by intrinsic and extrinsic defects in the solid (e.g., anion and cation vacancies, to form new photoinduced defects (F-type and V-type color centers) that alter the absorption spectrum of the solid by increasing extrinsic absorption. In this regard, absorption of light by these photoinduced defects is red-shifted compared to the original extrinsic absorption of the solid. Charge carriers that reach the surface of the solid by diffusion and/or drift can participate in interfacial charge transfer (redox) processes with pre-adsorbed species on the surface or with molecules in the gaseous phase.

#### 5.1.3. Photocatalysis of Organic Compounds

In general, the adsorption of chemical species at surfaces, an oxide surface in particular, modifies the electronic structure of both the surface and adsorbate, generating new electronic states from this interaction. In the case of weak interactions (physisorption), the adsorbed molecule maintains its atomic structure, and consequently the electronic structure is only slightly modified. In other cases, the geometry of the adsorbed molecule (or metal particle) is heavily modified by the interaction with the surface with a subsequent relevant change in the electronic structure. The adsoption properties of simple organic adsorbates on titania surfaces have been the subject of numerous computational studies [241]. Both clean surfaces and edge or corner sites of nanoparticles have been studied [241]. Understanding the structure, energetics and dynamics of organic molecules on various titania surfaces is of great interest. This section emphasizes the interaction of organic molecules like carboxylic acid, alcohol, and aldehydes.

The detailed mechanism of photocatalysis of organic molecules is well documented and described in Section 2. It has been theoretically shown that during photocatalysis by titania, most organic molecules can be directly oxidized by trapped holes, while the oxidation of some organic molecules with low HOMO energies progresses mainly through indirect oxidation by hydroxyl radicals [277]. Needless to say, water molecules are the most fundamental adsorbent on a titania surface. Some experimental data reported that a molecularly adsorbed form is dominant at low temperatures and that the dissociated form only occurs when the surface defects are mediated [278]. On the other hand, theoretical calculations have predicted both molecular and dissociated adsorption [279]. After a careful convergence check and controversy, it turns out that the preferred form of adsorbed water on titania relies on the choice of the DFT functional [280]. The hydroxyl radical has been proposed as the important active species on a titania surface. Shapavalov *et al.* using quantum chemistry method [281] discussed the formation of the OH^•^ radicals from adsorbed water molecules. The model intakes water molecules adsorbed on a cluster model of a titania (110) surface, in either a molecular or dissociated form. The electronic ground state and excited states were variationally calculated using the configuration interaction (CI) method [281]. From the results, the electron density between the electronic ground and excited states was compared, and the depletion of electron density on the water was found for the excited states, in both the molecular and dissociated forms. Shapavalov *et al.* [281] concluded that this feature would lead to the production of OH^•^ radicals, especially for the dissociated case. The transferred electron density appeared on the titanium atom in the third layer. Shapavalov *et al.* [281] related these findings to the proposed mechanism where electron holes migrate to the surface and trigger the production of radical species.

Carboxylic acid adsorption on titania surfaces is of great interest largely because carboxylic acids are a common anchoring group for photosensitizers and other functional molecules on titania. The adsorption of HCOOH on the surfaces of TiO_2_-rutileparticularly on the most stable (110) surface has been extensively studied, both theoretically and experimentally [75,276,282,283]. On oxidized rutile (110), it was established that HCOOH dissociates in two different modes [284]. In acidic dissociation, the OH bond is cleaved, resulting in a formate ion and a proton. The basic cleavage involves the breaking of the CO bond and the formation of HCO^+^ and OH^−^. Even though the basic dissociation is more likely in the gas phase, the acidic dissociation is much more favorable on the titania surface owing to the strong bidentate bridging between the resulting formate ion and the semiconductor surface [285]. Monodentate coordination of formic acid species hydrogen bonded to a surface two-fold coordinated oxygen is preferred, which is rather compatible with experiments of gaseous formic acid adsorption on anatase [286]. Moreover, since a dissociated bridging bidentate geometry is known to be the most stable for HCOOH on rutile (110), literature has confirmed the essential role of surface structure in determining the adsorption mode of a molecule [286]. The binding energy for the formic acid is significantly larger than that of water [286]. The bidentate bridging configuration is a more stable form for dissociative binding than the monodentate configuration. The formate ion creates a bidentate bridging structure both on wet and dry surfaces. On the hydrated anatase (101) surface, HCOOH forms an inner sphere adsorption complex, staying inside the first water layer and attaching directly to the surface.

Benzoic acid adsorption on rutile (110) and anatase (101) surfaces has been investigated to study its effect on the density of states of titania slabs, using different DFT methods [274]. Localized or plane-wave basis sets and all-electron or pseudopotential treatment were considered, using CRYSTAL06, SIESTA, and Quantum ESPRESSO packages [249]. A very good qualitative agreement and fairly good quantitative agreement between geometries, band gaps, and surface energies was obtained using different computational approach and DFT exchange-correlation functionals [274]. The density of states of rutile (110) and anatase (101) surfaces, show that conduction band edge states are localized away from the surface of the slabs; however, as the density of states increases to an appreciable number, the local density of states becomes increasingly spread evenly throughout the cell. The valence band edge is a mixture of the bulk and surface states; it is dominated by the fixed atoms’ states in constrained slabs. The band gap determined with B3LYP functional was larger compared to the GGA PBE and closer to experimental values. It was reported that the adsorption of benzoic acid has little effect on the electronic structure of anatase (101) slabs when the molecule is adsorbed both in the dissociated form and in the more stable nondissociated molecular form. The effect of the adsorbate on the density of states of rutile (110) slabs is more pronounced. Specifically, adsorption of benzoic acid increases the band gap of odd-layered rutile (110) slabs by raising the energies of subsurface Ti atoms states and thus raising the conduction band. This effect is much stronger in odd-than in even-layered slabs and decreases as the slab thickness increases, suggesting that slabs for modeling the rutile (110) surface should have at least four layers. The main difference in the B3LYP and PBE descriptions of TiO_2_ with adsorbed benzoic acid is that the benzoic acid HOMO appears slightly above the valence band edge in B3LYP (whereas it is at or below the valence band edge in PBE); however, this does not cause localization of TiO_2_ electronic states and does not change the structure of the bands.

**Figure 14:**
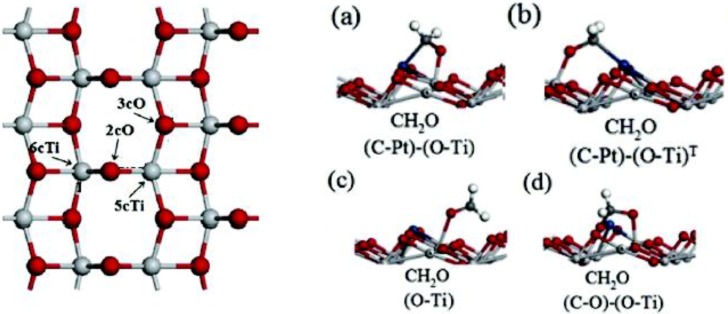
The stable adsorption structures of intermediates involved in CH_2_O oxidation on Pt/TiO_2_. Figure adapted from [287].

Formaldehyde, a widely used chemical in manufacturing industries, has been regarded as a toxic source as a volatile organic compound. Thus, removing formaldehyde has stimulated extensive explorations from both the experimental and theoretical points of view. The elimination mechanisms on the adsorption and dissociation of formaldehyde have been investigated [287]. Recently, Pt supported on anatase (Pt/TiO_2_) was found as one of the most active and promising catalysts for the catalytic oxidation of formaldehyde, which can work effectively without forming harmful byproducts even at room temperature. A recent DFT study by Li *et al.* [287] using the exchange-correlation functional of GGA-PW91 approximation, performed with the program package DMol3 in Materials Studio of Accelrys Inc. The perfect TiO_2_ (101) surface with a periodic (2 × 3) unit cell involving four Ti and eight O layers was used as the substrate. Usually, formaldehyde adsorbs on catalyst surface via the carbonyl oxygen at the Lewis acid sites (surface Ti ion in TiO_2_), which facilitates the attack of the nucleophilic species to the carbonyl carbon. Four stable adsorption configurations of CH_2_O on Pt/TiO_2_ are found (Figure 14): (a) (C–Pt)–(O–Ti), bridge adsorption between Pt and 5cTi on the same terrace; (b) (C–Pt)–(O–Ti) T, adsorption at step involving the Pt and a lower terrace 5cTi atoms; (c) (O–Ti), top adsorption over 5cTi; (d) (C–O)–(O–Ti), bridge adsorption between 2cO and 5cTi, similar to the configuration of CH_2_O adsorbed on ceria (Figure 14 for coordination notations 3cO, 2cO, 6cTi, 5cTi).

The most stable adsorption mode is (C–Pt)–(O–Ti) with E_ads_ = 1.34 eV. The catalytic oxidation of formic acid has been studied by four possible pathways, including direct dehydrogenation, dehydrogenation and oxidation into CH_2_O_2_ assisted by O^•^ and OH^•^. The intermediates of CH_2_O_2_, CHO_2_, CHO and CO_2_ prefer to adsorb at the Pt-5cTi bridge site, indicating that the Pt-5cTi bridge site is an essential active site. O^•^ has significant promotion effects on the C-H bond scission for CH_2_O and CH_2_O_2_ by weakening interaction of the adsorbates with the substrate in the initial state as well as dehydrogenated fragment. With departing H^•^ in the transition state, the structural deformation of the adsorbates when going from initial state to transition state is influenced. OH^•^ plays a same role by influencing interactions of adsorbates with substrate in the initial state as well as departing H^•^ atom and dehydrogenated fragment with substrate in the transition state. On the clean Pt/TiO_2_ surface, formaldehyde prefers successive dehydrogenation to CO along the route of CH_2_O/CHO/CO. In the presence of oxygen, formaldehyde would be oxidized to CO_2_ mainly along the reaction pathway of CH_2_O/CH_2_O_2_/CHO_2_/CO_2_, and CH_2_O/CH_2_O_2_/CHO_2_/CHO/CO/CO_2_ is a minor pathway. In this process, formate is an abundant surface species for its relatively low formation barrier and high decomposition barrier.

Volatile chlorinated organic compound such as trichloroethylene (TCE) has been widely used as an industrial solvent for degreasing of metals and for dry cleaning. Few studies have reported the possibility of interference of hydroxyl radicals on the TiO_2_ surface and initiate the photocatalytic degradation of the chlorinated compound [288]. With the help of frontier molecular orbital theory Yamazaki-Nishida *et al.* [288] showed that the OH^•^ radical attacks the CHCl side of TCE molecule forming a reactive intermediate that subsequently generated monochloroacetyl chloride which reacts with H_2_O to form monochloroacetic acid, the final product. However, in the heterogeneous system, TCE adsorbs on the surface of TiO_2_ whose d-orbitals can effectively interact with the p-orbitals of the carbon at the CHCl side. Therefore, the addition of the OH radical is possible only at the CC1_2_ side. The present study also indicates that a water molecule plays an another role for the reaction mechanism; it assists the tautomerism of 1,2-dichloroethenol by lowering the activation energy by 21.5 kcal/mol.

#### 5.1.4. Dye-Sensitized Solar Cell

Light is harvested by a large surface area of dyes grafted on the surface of a wide bandgap semiconductor (such as TiO_2_ or ZnO) in contact with an electrolyte (typically I_3_^–^/I^–^ in an organic solvent) and closed by a counter electrode, usually made of Pt. The elementary steps involved can be thus represented as follows [289]:

dye + hν → dye*
(32)

dye* → dye^+^ + e^−^ (33)

2dye^+^ + 3I^−^ → 2dye + I_3_^−^ (34)

I_3_^−^ + 2e_Pt_^−^ → 3I^−^ (35)

Upon absorption of light, dye molecules promoted in an electronically excited state (Equation 32), inject electrons into the conduction band of the semiconductor (Equation 33). Oxidized dyes are regenerated by a reducing agent of the electrolyte (usually I^–^), thus converted to the corresponding oxidized species (I_3_^–^, Equation 34). This latter is subsequently reduced at the counter-electrode (Equation 35). The overall charge carrier motion gives rise to the macroscopic photocurrent. As a consequence of the photoinduced electron transfer from the dye to the semiconductor, the electronic density increases in the oxide, giving rise to an electrochemical potential difference (*i.e*., a voltage) between the semiconductor and the electrolyte.

**Figure 15:**
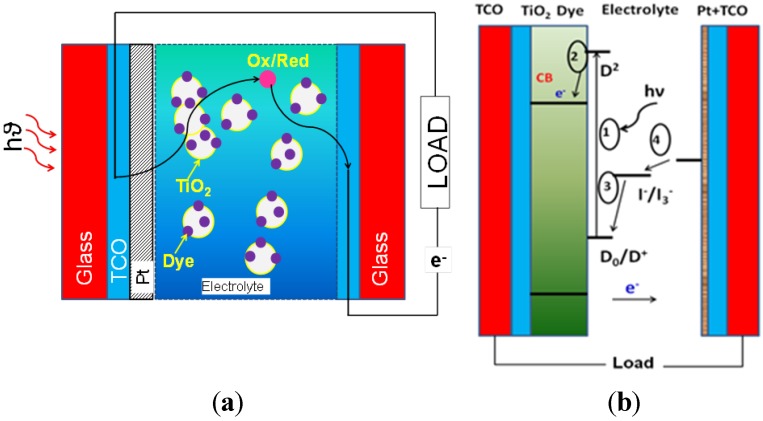
Cross-sectional view of dye-sensitized solar cells (DSSC) (**a**) schematic structure; and (**b**) working principles. TCO stands for transparent conducting oxide. (Figure adapted from Reference [289]).

The conversion of solar energy to electric current in DSSCs via photoexcitation of the chromophore molecule from its ground state (HOMO) which is located energetically within the semiconductor band gap, to an excited LUMO state of the chromophore that is resonant with the TiO_2_ conduction band (CB) (Figure 15). After the excitation, an electron is transferred from the chromophore to the semiconductor surface, typically on an ultrafast time scale, τ*_i_* (Figure 13). The injection competes with intramolecular relaxation to lower energy excited states, such as the triplet states of the semiconducting surface [290,291] or back to the ground state (Figure 13). Following this electron transfer, the electron diffuses into the bulk, τ*_d_*, and simultaneously relaxing to the bottom of the CB and losing its energy to vibrations, τ*_v_* in Figure 13. The cell circuit is completed by movement of the electrons to the electrode that is attached to the semiconductor, and subsequently coming back to the chromophore ground state. Occasionally, the electron remains trapped at the surface. The trapping of the electron is most likely to occur if the relaxation inside the CB occurs faster than the delocalization into the bulk, and if the surface contains many defects or unsaturated bonds that support surface states. However, surface trapping can also be important even if the electron is able to diffuse into the bulk. In order to optimize light harvesting and maximize charge separation, the semiconductor is designed to have a high surface area with multiple chromophore adsorption sites. For instance, in the case of TiO_2_ nanoparticle semiconductor, the injected electron is never far from the surface and can easily return to the surface before it reaches the electrode. The position of the electron relative to the surface determines whether it will reach the electrode or whether it will recombine (τ*_r_*) with the positive charge remaining on the chromophore after the injection or by transferring onto the electrolyte mediator. The mediator is located near the surface, since it brings electrons from the counter electrode in order to regenerate the neutral chromophore.

Certainly, the relative yields and rates of these processes influence the efficiency of the cell. If the photoexcited electron decays to a low-energy chromophore state on a faster time scale than the injection, or if the recombination at the surface is faster than the delocalization into the bulk, there will be little current. Similarly, if an electron that has already reached the bulk returns to the surface and recombines with the chromophore or electrolyte, the current will be reduced. It is also a concern that electron relaxation to the band edge can decrease cell voltage. Developing a theoretical understanding of each of these processes is vital to the efficient improvement of solar devices. The electron injection is typically faster than the intramolecular relaxation to the ground electronic state; the latter rarely affects the cell performance. The role of the electron back transfer (back-ET), however, is particularly important. The relaxation and delocalization of the electron within the TiO_2_ CB influences the back-ET rate and are also critical processes to understand.

#### 5.1.5. Chromophore Binding to Titania Surface

The intrinsic structural and electronic properties required for a dye to generate a photocurrent upon adsorption on an oxide surface can be defined and computed on the following basis [266]:

- the anchoring group should be present on the electron-accepting group;
- the HOMO and LUMO of the dye should be energetically computed above the VB edge and the CB edge of the oxide, respectively;
- the dye absorption spectrum should match the solar spectrum;
- high intensity transition in the photoconversion;
- the fluorescence lifetime should be sufficiently high (above the nanosecond time scale) to allow an electronic injection to the semiconductor from the excited state of the dye before quenching of the excited state by a radiative decay.

The chromophores are typically bound to the TiO_2_ surface with oxygen containing substituents, such as hydroxy, carboxy, and phosphoric acid groups. Binding via the hydroxy group results in a very strong chromophore-semiconductor coupling because only a single oxygen atom separates the chromophore from the semiconductor. The coupling decreases with carboxyl binding, and it decreases even further with binding through the phosphoric acid group. The interaction of small organic anchoring groups such as carboxylic acids and alcohols, as the binding sites of dye molecules with TiO_2_ surfaces has been investigated [241]. Presence of water also affects the process to a large extent.

Considering carboxylic acid as anchoring groups, Bi-isonicotinic acid has been studied theoretically and experimentally [292,293]. Bi-isonicotinic acid can form up to four chemical bonds with the rutile (110) surface. Semiempirical calculations on bi-isonicotinic acid adsorbed to rutile (110) surfaces [294] predicted the most stable structure which has a twist around the molecular axis with the pyridine rings tilted in opposite directions. DFT studys of adsorption of bi-isonicotinic acid on rutile (110) [292] confirmed that the most stable structure was a bidentate bridge with a large twist between the isonicotinic acid groups. Comparing the adsorption energy of two isonicotinic acids with that of a single bi-isonicotinic acid, the authors found there was a 40% destabilization that resulted from the adsorbate strain in the second structure. The periodic semiempirical calculations of bi-isonicotinic acid on anatase (101) [294] showed the dissociated bi-isonicotinic acid protons were bound to the surface oxygens.

With respect to alcohol anchoring groups, catechol is one of the smallest chromophores, which have been studied in a dye/semiconductor system. The catechol/TiO_2_ interface exhibits many interesting features in both its electronic structure and its electron transfer (ET) dynamics. As a result of a strong electronic interaction and a direct charge transfer from catechol to the semiconductor surface, semiconductor surfaces with catechol causes a strong red shift in the spectrum [268]. A transition between this dye orbital and energy levels with strong contributions from Ti 3d atomic orbitals near the point of attachment was a strong contributor to the low-energy band. The accepting orbitals near the CB edge, including the LUMO, had no contribution from the catechol, so the charge transfer was complete.

Alizarin is closely related to catechol and prefers to bind to the semiconductor through two neighboring hydroxyl groups. Alizarin and Quinizarin, on TiO_2_ was studied using *ab initio* method previously investigated by other groups [265,295]. Alizarin being a larger molecule than catechol, which contains an extended π-electron system, has smaller excitation energy comparatively. Both alizarin and quinizarin have excited states close to the edge of the TiO_2_ CB, giving rise to interesting features in electronic structure and dynamics. The optical activity for the charge-transfer excitations in the TiO_2_-bound alizarin is significantly smaller than in the catechol system, even though the chromophore semiconductor coupling is exactly the same. The difference stems from the large amount of mixing between the molecular excited π-orbital in alizarin compared to catechol that provides the optical activity to the charge-transfer states and the Ti orbitals.

Chromophores comprised of a transition metal, most often Ru, and several ligands are the standard in photovoltaic applications [243] as the metal center can easily switch between its oxidized and reduced states. The smaller ligands participate in the regeneration of the neutral chromophore from the cation after photoinduced ET. The ligands assist in transferring the electron from a redox mediator in solution to the ground state of the dye. The HOMOs of the transition metal based chromophores are primarily composed of the d-orbitals of the metal, whereas the LUMOs are localized on the organic ligands [240,266].

Photoexcitation induces intrachromophore charge transfer, shifting the electron density toward the surface. The full system containing the metal, the ligands and a TiO_2_ surface has not been detailed by an atomistic computational study. The isolated chromophores have been investigated rather comprehensively, however, both by semi-empirical and *ab initio* approaches [243,296]. These studies address several issues: (a) the origin of the bands in the chromophore optical absorption spectra that ideally cover a wide range of wavelengths; (b) the choice and optimization of the ligands in order to achieve both strong, broad absorption and efficient electron injection; (c) the solvent effects, including dye protonation and deprotonation by a change of pH; and (d) the triplet states.

With regard to dyes, the best photoconversion efficiencies are obtained with ruthenium- and osmium-based dyes for TiO_2_-based cells, while organic dyes such as eosin-Y and indolines are common dyes used in ZnO-based cells, especially due to their low costs [243]. Most of the organic dyes used are generally “push-pull” architectures made of the covalent assembly of (i) an electron-donating group and (ii) an electron-withdrawing group. In this case, the excited state responsible for the injection into the conduction band of the semiconductor is generated by a charge transfer (CT) transition from the donor to the acceptor group. On the other hand, in the case of Ru or Os complexes, the excited state basically corresponds to a metal-to-ligand CT transition [240]. The main electronic requirement for semiconducting DSSCs application is related to the position of the band edges. For isolated chromophore dyes, several studies report the electronic structure theory of TiO_2_ and ZnO semiconducting devices [243].

The electronic structure and spectra of cisbis(4,4′-dicarboxy-2,2′-bipyridine)bis(isothiocyanato) ruthenium(II) a common a transition-metal sensitizer chromophore, was computed using semi-empirical INDO method. The results matched the experimental spectra quite well [297]. The atomic orbitals centered on the isothiocyanato (NCS–) ligands contributed to the highest energy valence orbital depopulated by the photon absorption. This orbital plays a key role in accepting an electron from the mediator in DSSCs. Because the NCS–ligands are directed away from the semiconductor and toward the solution, this type of dye should be particularly well suited to reduction from the electrolyte.

Black dye is a trithiocyanato(4,4′,4′′-tricarboxy-2,2′:6′,2′-tripyridine)ruthenium(II) complex is one of the most efficient light absorbers that can be used in solar cells [243]. The monoprotonated form gives the best results in experimental studies of black dyes adsorbed to TiO_2_. The electronic-structure calculations showed strong absorption over the whole visible spectrum, as well as in the IR. The ligand-to-ligand charge-transfer states dominated in the low-energy part of the spectrum. These states corresponded to an electron on the terpyridine ligand and a hole on the NCS–ligands. The higher-energy metal-to-ligand states also had an electron on the terpyridine ligand, but the hole was located on the metal center [243].

### 5.2. Electron Transfer Process

The theoretical study of interfacial electron transfer (ET) reactions requires a quantum mechanical description for the overall processes including a characterization of the electronic structure of the system as well as a simulation of the ET dynamics. The electron transfer dynamics should consider the coupling of nuclear degrees of freedom along with electronic. To this end, a variety of different methods have been developed and employed. The dynamics of electron injection at the dye-semiconductor interface has been studied by employing first-principles simulations [264,295,298,299,300] as well as models based on a parameterized Hamiltonian [300,301]. While the former class of methods typically uses an approximate classical treatment of the nuclear dynamics, the model-based approaches often allow a full quantum dynamical treatment.

The dynamics of photoinduced electron transfer processes at the dye–semiconductor interface has been studied with different approaches and methods. Thoss and co-workers developed an *ab initio* based method, called the multilayer multiconfiguration time-dependent Hartree (ML-MCTDH) method [295,296]. This method employs a representation of the Hamiltonian in localized donor and acceptor states. The donor and acceptor states as well as other parameters of the ET Hamiltonian are determined using a partitioning method based on electronic structure calculations. On the basis of this modeling, the quantum dynamics of the ET process is simulated using the ML-MCTDH method [295].

Prezhdo’s group used nonadiabatic molecular dynamics to simulate electron transfer on time scales of up to tens of femtoseconds to distinguish between adiabatic and nonadiabatic electron transfer pathways. For example, electron dynamics and electron transfer rates at the alizarine/TiO_2_ interface were studied [264,265,266]. However, even if an *ab initio* description of the interface is appealing, this kind of time-resolved simulation is still rare and computationally demanding. Batista and co-workers used *ab initio* molecular dynamics (within DFT) and adapted model Hamiltonian derived using the semi-empirical extended Hückel approach to describe the excited states and propagate the wave function in time, for representative nuclear configurations. In this way, they modeled the dye–semiconductor electron transfer and subsequent charge delocalization in TiO_2_ crystals [263]. May and co-workers [302] studied the heterogeneous electron transfer for the TiO_2_–perylene derivatives system using a diabatic-like separation of the whole system into molecular and semiconductor states. They also considered the ground and first excited state for the dye together with a large number of states in the conduction band of the semiconductor. This model is based on experimental results since the Hamiltonian features parameters that are fitted to measured spectra. Linear response real-time (LR-RT) TDDFT approach coupled with equation of motion-coupled cluster (EOMCC) has been used to study the excitation process. Most of these models require a simulation of a complete semiconductor–chromophore system and are therefore not suitable for the screening of properties of large numbers of chromophores. The modeling of a complex system like DSSC cannot be limited to the atomic level processes, but requires the study of the charge transport across the full device with suitable mesoscale methods. The charge transport in DSSC has been studied using a continuous-time random walk method by Nelson *et al.* [303,304] focusing on the movement of electrons through the semiconductor and identifying how this affects the various electron loss mechanisms. Walker *et al.* have also studied the electron transport properties of nanocrystalline TiO_2_ using a random walk Monte Carlo technique to determine how the morphology of the TiO_2_ and the concentration of trapping states affects the rate of electron transport [305]. These mesoscopic models may be able to use kinetic parameters determined from first principles to correlate microscopic kinetics with observed efficiency. *Ab initio* electron dynamics [300] simulations also confirms strong coupling between dye and TiO_2_ surface, favoring an ultrafast electron transfer process between them. From this study they also noticed an effect of cluster size on electron transfer time.

*Ab initio* time-domain atomistic simulations and investigations determine the rates and mechanisms of the electron injection [240]. Nonadiabatic (NA) molecular dynamics simulations in real time and at the atomistic level using a mixed quantum-classical approach that combines TDDFT with surface hoping (SH) in the Kohn-Sham (KS) representation. This method, unlike the Ehrenfest and classical path techniques used previously, maintains detailed balance and allows us to model electron relaxation. The simulation is done for the entire system that contains many electronic states that have shown the relaxation to the edge of the CB occurs on an ultrafast time scale, with the rate independent of the energy of the injected electron relative to the CB edge. This is possible because, as electrons progress down the band, they populate states that have strong coupling with band edge states. Large downward hops in energy between these states and the band edge states make a significant contribution to the electron dynamics.

This methodology reports a competition between NA and adiabatic types of ultrafast transfer [243]. Adiabatic ET is mediated by a strong coupling between the chromophore and the semiconductor and during the transfer, the electron remains in the same adiabatic state that changes its localization from the dye to the semiconductor. In NA ET the electron quantum mechanically hops (tunnels) between adiabatic states. At any particular nuclear configuration, the NA movement of charge from the dye to the semiconductor can occur and this is particularly important when the dye-semiconductor coupling is weak. The NA electron transfer relies on a high density of states (DOS) in the CB. As the DOS increases with energy, an increase of the chromophore excited-state energy relative to the CB edge will accelerate the NA transfer. Simultaneously, the photoexcitation energy and solar cell voltage will be lost due to the relaxation of the injected electron to the bottom of the CB. In the event of NA ET it is also vital to minimize chromophore intramolecular vibrational relaxation, which lowers the chromophore energy and thereby the accessible DOS. With the increasing distance between the donor and acceptor species, the rate of NA ET will decrease exponentially. However, the adiabatic ET requires strong donor-acceptor coupling but depends much less on the density of acceptor states. As adiabatic transfer requires certain fluctuation in the energy that can bring the system to the transition state, a fast exchange of energy between vibrational modes of the chromophore will increase the likelihood of adiabatic ET.

**Figure 16:**
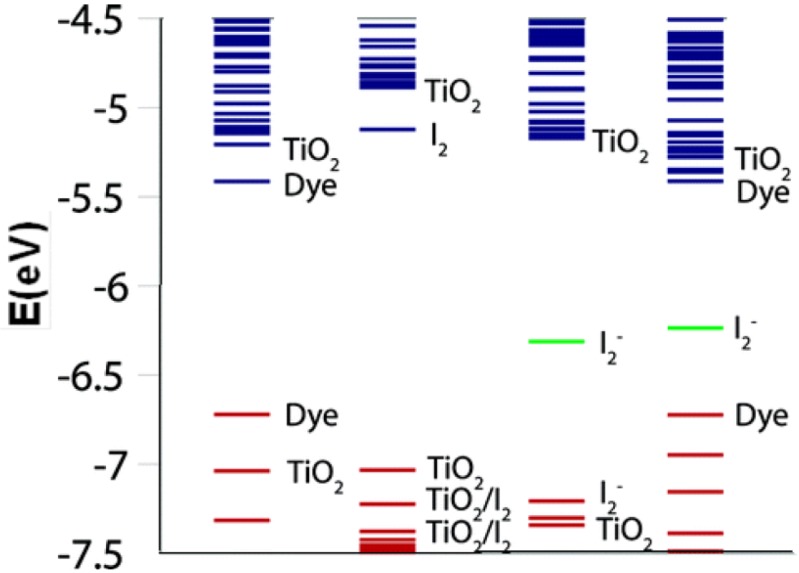
Energy diagrams involving the key states in the alizarin/TiO_2_, I_2_/TiO_2_, I_2_-/TiO_2_, and alizarin/I_2_-/TiO_2_ systems with optimized geometries. The doubly and singly occupied and vacant states are denoted by red, green, and blue bars (gray, light gray, and black in black-and-white), respectively. The alizarin excited-state is located slightly below the TiO_2_ CB, while the alizarin ground state is well inside the band gap and is closer to the TiO_2_ VB. The lowest energy vacant state of I_2_-neutral is also slightly below the TiO_2_ CB edge, while the I_2_ ground state is inside the VB. The I_2_-anion singly occupied state is in the middle of the TiO_2_ band gap. The TiO_2_ surface state energies are notably perturbed in the combined system, such that the CB edge moves down, closer to the alizarin excited state. (Figure adapted from Reference [240]).

Duncan *et al.* [240] studied TiO_2_-alizarin system using non adiabatic molecular dynamics, and found that in low-temperature simulations, the chromophore was attached to the semiconductor via a carboxylic acid bridge. The photoexcited state of the chromophore was well within the CB and a NA mechanism dominated (Figure 16) [243,264,266].

The adiabatic mechanism was the predominant process for the majority of ET, both for injection deep within the band occurring through the carboxylic acid bridge and for injection at the band edge occurring through a hydroxyl bridge. Contrary to NA ET, adiabatic ET can occur even on an ultrafast time scale even at the band edge, where the DOS is much smaller. This finding was especially helpful in the design of cells with higher voltages, as the voltage lost by the electron relaxation in the CB will be much smaller. In line with earlier results, the delocalization of the electron from the surface into the bulk takes on the order of ~100 fs. It was also determined that the back transfer of the electron trapped near the surface to the electrolyte can occur on a picosecond time scale [265]. This period was based on the assumption that the mediator can closely approach the surface, which is reasonable since the electrolyte is attracted to the oxidized chromophore and must remain near the surface in order to reduce the chromophore. Simulations that model the back-ET from the semiconductor to the chromophore occur on a picosecond or longer time scale, in agreement with experimental values. In the presence of the electrolyte mediator, the back transfer from the semiconductor to the dye occurs on a similar time scale. Although the mediator is rather close to the dye and the surface, the electron often transfers directly from the CB edge back to the dye. An electrolyte molecule can approach the surface close to the attached chromophore and then transfer an electron onto the chromophore, preparing it for the next photovoltaic cycle. The simulation indicated that the electrolyte-chromophore coupling could occur both through space and with the help of the surface states. Once the electrolyte species passes its charge to the chromophore, it is no longer strongly attracted to the surface and will diffuse away. The back-ET is also rather fast and it proceeds via the NA mechanism, since the energy gap between the bottom of the TiO_2_ CB and the chromophore ground state is large. The high back-ET rate observed in the TiO_2_-alizarin system is due to strong NA coupling. The dependence of the NA coupling on the electronic overlap suggests choosing chromophores with ground states localized away from the surface can reduce that back-ET. Surprisingly, the rate of back-ET is not particularly sensitive to the exact value of the energy gap between the TiO_2_ CB and the chromophore ground state, as long as the gap remains large.

**Figure 17:**
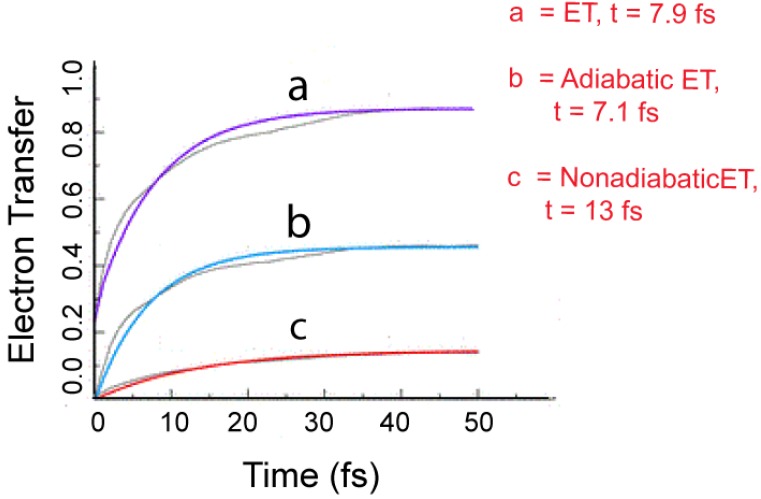
The electron-transfer (ET) dynamics averaged over ensembles of initial conditions for alizarin systems at room temperature. (Figure adapted from Reference [243]).

For the ET dynamics of the alizarin system, the total ET occurred on a sub-10-fs timescale. There is a substantial amount of charge transfer during photoexcitation (25%) where the adiabatic mechanism had a faster rate (7.1 fs) than the nonadiabatic (NA) mechanism (13 fs). Moreover, the adiabatic ET was a much larger fraction of the total transfer. Further ET features were revealed by separately averaging over the high- and low-energy initial conditions corresponding to the photoexcited states inside and below the TiO_2_ CB. Averaged over just the higher-energy initial states, both the total and adiabatic ET were faster, 3.6 and 3.2 fs, respectively. The adiabatic mechanism was more dominant relative to the NA ET as there was a larger amount of initial charge transfer. The overall shape of the ET curves for the higher-energy initial conditions was similar to the average data (Figure 17).

Transition-metal-based chromophores that are most commonly used in DSSC take advantage of the dependence of the NA coupling on both the electronic overlap and the vibrational velocity. In such chromophores, the ground state is localized away from the surface and on the transition metal. The latter is a heavy element, and therefore the frequency of the vibrational motions that couple the ground state localized on the transition metal is low.

**Figure 18:**
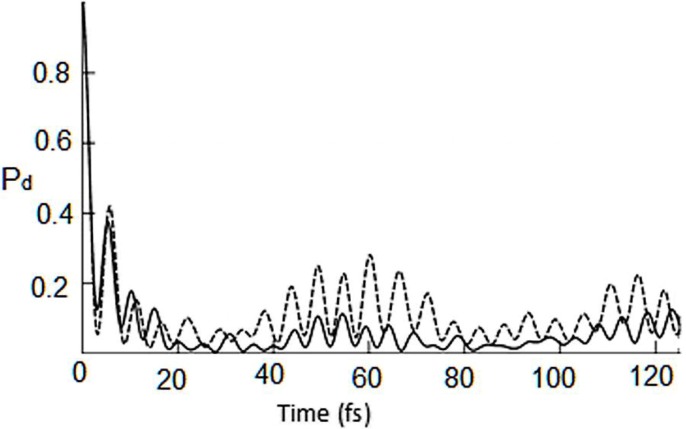
Population dynamics of the donor state after photoexcitation. Shown are results obtained for (**a**) the finite (TiO_2_)54 substrate and (**b**) the model of both results with vibronic coupling (solid lines) and without vibronic coupling (dashed lines) are depicted Figure adapted from Reference [306].

The interfacial ET processes, considering photoinduced electron injection in the dye-semiconductor system was studied for a coumarin 343 (C343)-TiO_2_ and alizarin-TiO_2_ system using ML-MCTDH method [295,306]. The ET dynamics in the alizarin-TiO_2_ shows the result of the simulation of the population of donor state after photoexcitation (solid line, Figure 18). The initial decay of population of the donor state reveals an ultrafast injection of the electron from the donor state localized at the chromophore into the quasicontinuum of acceptor states.

Localized in the TiO_2_ substrate on a time scale of a few femtoseconds, due to electronic coherence, they noticed a difference in behavior with the results of a purely electronic calculation (dashed line), where the nuclear degrees of freedom are frozen at their equilibrium geometry. Also, analysis of the electronic-vibrational coupling in the ET process shows that the coupling to the vibrational modes of the chromophore results in a somewhat slower injection dynamics. However, due to the ultrafast time scale of the ET process and the relatively small reorganization energy (0.155 eV), the overall effect of electronic-vibrational coupling in the alizarin-TiO_2_ system is rather small. Also the electron injection dynamics cannot be characterized by a single rate constant, due to the coherent oscillatory character of the dynamics. However, the overall time scale of about 5–10 fs as obtained in the simulations agrees well with the experimental result of 6 fs. Also their finding suggests that the electron injection process proceeds with a two-step mechanism, which involves an intermediate state localized at the dye-semiconductor interface. The strong coupling between the donor and intermediate states results in coherent electronic motion, which is damped due to the interaction with the substrate.

Barista and coworkers used *ab initio* DFT molecular dynamics simulations together with quantum dynamics calculations of electronic relaxation to investigate the excited state interfacial electron-transfer dynamics in sensitized semiconductors [263]. They studied the ultrafast electronic relaxation in the semiconducting-anchor material, with a reliable description of the electronic couplings between the discrete molecular states in the anchored molecule and the dense manifold of highly delocalized electronic levels in the semiconductor. As the time scales associated with the electronic relaxation are much faster than the nuclear motion, they assumed a decoupling of the electronic and nuclear dynamics to investigate the excited-state electron transfer at the detailed molecular level. They obtained representative nuclear configurations according to *ab initio* DFT molecular dynamics simulations. The time-dependent electronic wave function is propagated according to a model Hamiltonian gained from the semi-empirical extended Hückel (EH) approach. For an example, in a catechol/TiO_2_-anatase nanostructure system, their results show that the reaction mechanisms as well as the characteristic times for electron injection are very sensitive to the symmetry of the electronic state initially populated in the adsorbate molecule. This is due to the fact that the electronic couplings drive interfacial reaction dynamics between the initially populated electronic state in the molecular adsorbate and the electronic states of similar energy in the conduction band of the semiconductor. The electron injection from the catechol-LUMO is essentially mediated by a primary electron-transfer event that localizes the injected charge in the Ti^4+^ surface ions next to the photoexcited adsorbate molecule within the first 5 fs of dynamics. The photoexcitation followed by injection dynamics to the catechol-LUMO was directed by the electronic couplings between the catechol-LUMO and the d-orbital of the nearest Ti^4+^ ion. This process was followed by charge separation and delocalization (*i.e*., carrier relaxation) through the anatase crystal. It was noticed that both the initial injection and the subsequent delocalization processes are faster than the electron injection from the catechol-LUMO. Furthermore, carrier relaxation after photoexcitation to the catechol-(LUMO+1) is significantly different from the charge delocalization process. Here, carrier relaxation involves charge diffusion along the semiconductor surface before the injected charge separates from the surface.

### 5.3. Future Aspects

At first sight, metal oxide surfaces like TiO_2_ could appear to be a rather exotic field, and has become a field of enormous interest because of the direct relationship to photocatalysis and DSSC, the core of two relevant technologies of paramount importance for a sustainable society. The input from technology has been a driving force for the basic research, for both theory and experiments. Theoretical methods together with appropriate surface models are nowadays capable of treating very large oxide systems with an increasing predictive power, especially for the ground-state properties and chemical reactivity. Nevertheless, one must warn that in some cases the situation is less clear and even the nature of the ground state constitutes a challenge for the present methods. This situation is especially the situation encountered when several open shells are involved and the electronic states arising from the different possible spin couplings are near degenerate. In any case, the progress in DFT and TD-DFT methods, which are nowadays applicable to cluster or periodic surface models, has paved the way for the study of more realistic systems. The spectroscopic characterization of point defects at oxide surfaces and the theoretical study of the low-lying states of probe molecules at oxide surfaces can nowadays be studied in an almost routine way.

The TiO_2_ nanostructure interaction with molecular moieties is an excellent case study for elucidating the issues that arise when localized molecular species are combined with extended bulk materials. Such configurations have become increasingly common in recent years, as molecular and solid-state domains have converged wide application in different areas including photovoltaics, photo- and electrochemistry, molecular electronics, photography, detection tools, bioanalytical chemistry, and biomechanics. Analysis of the available theoretical results on the structure, electronic properties, and electron-vibrational dynamics in the organic/inorganic molecule-TiO_2_ interfacial systems indicates that the adsorbent creates a local perturbation within the extended TiO_2_ system. Most of the experimental data characterizing the effect that bulk TiO_2_ has on adsorbate molecules can be modeled and understood with relatively small-scale calculations that include moderate-sized portions of the semiconductor. A cluster representation of the semiconductor can often be sufficient, and sometimes even the crudest few-atom representation of TiO_2_ captures the essential phenomena. This conclusion holds for both ground and low-energy excited states of molecular system. However, the size of the molecule-semiconductor interaction region increases with energy.

Real-time modeling of electron-vibrational dynamics is particularly valuable for understanding the interfacial electron injection because it occurs on ultrafast timescales and shows a variety of individual injection events with well-defined dynamical features that cannot be made apparent by an average rate description. Such simulations are rare, but computationally demanding state-of-the-art techniques for the dynamics simulations are currently being developed. As the research progresses, larger systems and longer timescales will become accessible, allowing one to probe more examples and finer details of the interfacial ET and to study different surfaces, surface defects, bridges, temperature, and solvent dependence. One can anticipate that real-time dynamics simulations in the near future will address other important aspects of the interfacial ET, including the back-transfer from the surface onto the dye and the delocalization of the electron from the surface into the bulk. However, reaching this horizon, even if already in sight, will still require a continued effort in the forthcoming years. Little is known about the reactivity on the excited states and insight will require the development of new methods and techniques; the road is still to be built, but the destination is clear and known.

## 6. Concluding Remarks

To this end, a large variety of synthesis techniques have been adopted to improve the catalytic performance of metal oxide nanotube arrays formed by electrochemical anodization process in solar converting energy systems. Although several of the systems investigated are far from obtaining theoretical limits in solar energy conversion, further advances in conversion efficiency are progressing, however rather slowly. A large limiting factor in further progress is the development of a fundamental understanding of charge transfer dynamics and kinetics. It is envisaged that any further progress necessitates future investigations to couple theoretical modeling with experimental verification. Moreover, parallel development of system engineering is also required if a technical commercial process utilizing solar energy and these materials is to be realized.
